# Too threatened to try: how extinction risk drives research neglect in rays and skates

**DOI:** 10.1098/rspb.2026.2126

**Published:** 2026-11-04

**Authors:** Andrew J. Temple, Sophia Rosinski, Jesse E. M. Cochran, Faizan F. Khan, Lindsay Marshall, Michael L. Berumen

**Affiliations:** ^1^ Biological and Environmental Science and Engineering Division, King Abdullah University of Science and Technology, Thuwal, Saudi Arabia; ^2^ Computer, Electrical, and Mathematical Science and Engineering Division, King Abdullah University of Science and Technology, Thuwal, Saudi Arabia; ^3^ Stick Figure Fish Illustration, Peregian Beach, Australia

**Keywords:** causal inference, conservation, charisma bias, elasmobranchs, accessibility bias

## Abstract

Global biodiversity loss demands that research effort be directed towards species most at risk. Yet conservation science is biased by species' accessibility and perceived charisma, raising concerns that threatened species may be overlooked. We apply a structured causal modelling framework to 651 species of rays (superorder Batoidea), a highly threatened yet understudied lineage of cartilaginous fishes, to disentangle the drivers of research effort. We quantify the causal effects of taxonomy, extinction risk, accessibility (range size, depth, economic context, year of description) and charisma (body size, shape, patterning and colouration) on species-level research outputs from more than 7000 publications. Research effort was strongly taxonomically biased, driven by accessibility and charisma, with shallow depth, broad geographic ranges, large body size and distinctive morphologies attracting disproportionate attention. Extinction risk exerted a counterintuitive nonlinear effect: Critically Endangered and Endangered species received significantly fewer publications than Near Threatened and Vulnerable species and were comparable to Data Deficient species. This effect is consistent with a ‘Goldilocks’ zone in which species are threatened enough to justify research but not so threatened that it becomes prohibitively challenging. Systematic research bias away from the species most in need undermines conservation efforts. Realigning research priorities is essential to combating the biodiversity crisis.

## 1. Introduction

The biodiversity crisis is one of the most pressing environmental challenges of our time [1]. Species are declining at unprecedented rates owing to habitat destruction, overexploitation, climate change and pollution [2–5]. Scientific research plays a crucial role in identifying, understanding and managing the species most at risk. Yet, research underpinning conservation and management efforts is often skewed towards more charismatic or economically valuable species [6–9]. Such biases in research may result in lesser known but ecologically important and/or highly threatened species being overlooked, limiting the effectiveness of conservation interventions.

From a conservation prioritization standpoint, species at highest risk of extinction (i.e. those listed as Vulnerable, Endangered or Critically Endangered by the International Union for Conservation of Nature (IUCN)) would ideally receive the greatest research attention. Evidence for whether such prioritization occurs in practice is conflicting and so remains uncertain [8–13]. Although previous studies have shown various relationships between extinction risk and research outputs, these have been established through correlative approaches, rather than through the establishment of causality. Research outputs are influenced by additional variables, such as species' accessibility and perceived charisma, that also drive extinction risk and so may confound the relationship. As a result, correlations previously found between endangered status and research outputs may not reflect causal relationships.

Disentangling the direct and indirect effects of variables that drive both research effort and extinction risk is critical to establishing evidence-based conservation priorities. Doing so requires analytical approaches capable of moving beyond correlation [14] and which explicitly and appropriately account for confounding variables. For example, research effort may be influenced by the accessibility of species, such as the logistical difficulties of working on species inhabiting high altitudes or deep oceanic waters, and the financial capability to fund research. These logistical and financial elements also influence humanity's ability to exploit and manage species, thereby impacting conservation status [2,15,16]. Similarly, perceived aesthetic appeal, or ‘charisma’, may disproportionately draw research attention to species which are unusually large, vividly coloured, uniquely patterned or culturally significant [13,17–19]. Many of these traits are themselves linked to extinction risk. For example, larger species typically have lower maximum population growth rates and are therefore more vulnerable to overexploitation [20]. Understanding the causal interplay between these drivers is essential for disentangling the causal effects of extinction risk from the biases that influence species research prioritization.

Rays and skates (superorder Batoidea, hereafter ‘rays’), one of three major lineages within the class Chondrichthyes alongside sharks and chimaeras, provide a compelling case study for exploring how extinction risk, accessibility and charisma interact to shape research biases. Chondrichthyans are highly threatened, with 37% of assessed species threatened with extinction and average population declines approaching 50% [2,21,22]. Rays represent the most species-rich and most threatened subgroup, with over 650 described species, 41% of which are assessed as threatened with extinction [22]. They also contain the first documented marine fish extinction resulting from human pressures, the Java stingaree *Urolophus javanicus* [23]. Although chondrichthyan research has intensified in recent years and is generally well prioritized towards key anthropogenic threats [24], rays receive far less attention than sharks (superorder Selachimorpha) [10]. Rays are distributed circumglobally, ranging from riverine systems and coastal waters to some of the deepest parts of the oceans. They exhibit remarkable variation in body size, from the 7 m wingspan of the oceanic manta ray (*Mobula birostris*) to the 15 cm total length of the finless sleeper ray (*Temera hardwickii*) and they vary in both colouration and patterning.

Using rays as a case study, we investigate the causal effects of endangered status and key confounding variables related to accessibility and charisma on species-level research outputs. We hypothesize that research effort is causally driven by accessibility (depth, geographic range, gross domestic product, year of description), charisma (body size, shape, patterning, colouration) and conservation status, and that these drivers are themselves causally interrelated in ways that create confounding. By applying a structured causal modelling framework, which makes causal structure explicit, including hypotheses about which variables should not be conditioned on to avoid collider bias or overcontrol, we aim to identify the underlying drivers of research prioritization. In doing so, we seek to clarify how research effort is allocated, the extent to which it aligns with conservation need, and the implications of any mismatch in addressing the biodiversity crisis.

## 2. Material and methods

### (a) A causal framework for research effort

Following the structural causal modelling framework [14], we developed a directed acyclic graph (DAG) to represent our hypothesized causal structure linking species' extinction risk, accessibility and charisma to research outputs (figure 1). In a DAG, each variable is represented by a node, with edges indicating hypothesized causal relationships and their directionality. The absence of an edge denotes a hypothesized lack of direct causal influence, and no closed causal loops are permitted (acyclicity). The DAG thus makes the full causal structure explicit, including which variables are hypothesized to exert direct versus indirect effects on research effort, which pathways mediate relationships among accessibility, charisma, and conservation status variables, and which variables are hypothesized to have no causal influence. This contrasts with traditional statistical approaches, where associations among variables are often selected for based on statistical significance and/or model fit, and where causal assumptions remain only implicit in model specification. Beyond guiding which variables must be conditioned on to identify causal effects, the DAG also identifies variables that must be excluded from conditioning sets to avoid collider bias (which can induce spurious associations between variables where none exist) or over-control (where adjusting for mediators or descendant variables can obscure causal relationships). Justification for each hypothesized causal link in our DAG are provided (see supplementary material). While DAGs are widely used in other scientific fields, their use in ecology and conservation remains relatively new. Step-by-step guidance for their implementation in ecological studies is available [25,26]. The variables identified in the DAG are used to inform data gathering (see §2b). Further, the DAG provides the framework that guides our causal analyses, specifically how we structure our statistical models when assessing the causality of each variable on research output (see §2c).

**Figure 1.**
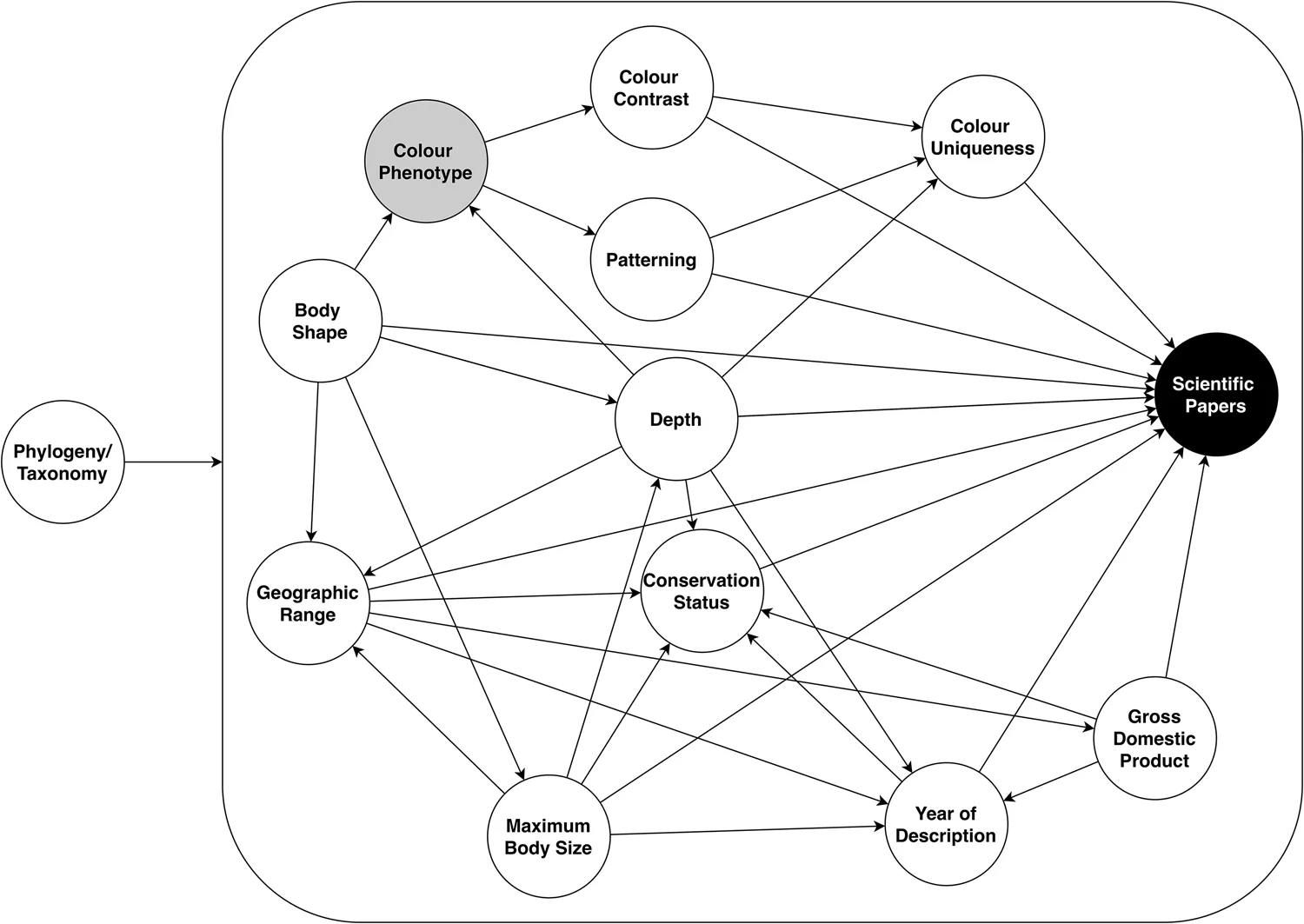
Proposed causal structure of variables driving research output in rays (superorder Batoidea), presented in the form of a directed acyclic graph (DAG). Variables include those proposed to affect species' accessibility (geographic range, depth, year of description and gross domestic product across species' range), perceived charisma (maximum body size, colouration contrast, colouration uniqueness, patterning and body shape), and the conservation status of species (IUCN Red List status). Colour phenotype is an unmeasured variable that drives contrast and patterning.

### (b) Data collection

Our study included all species of ray with current IUCN Red List Status assessments as of 01 July 2025 (IUCN v2025-1), totalling 651 species of the approximately 667 currently recognized [27]. For each species, we extracted the IUCN Red List status, synonyms and taxonomic authority (from which we extracted year of description), as well as minimum and maximum depth ranges. We also downloaded each species' geographic distribution shapefile [22] to calculate total distribution range and identify all countries with exclusive economic zones overlapping species' distribution range. Shapefiles were reprojected to the Mollweide Projection, after which two-dimensional area was calculated. Country-level gross domestic product (GDP) was sourced from the World Bank [28], and the median GDP from the last 10 years was calculated. Species-level GDP was calculated as the sum of median GDPs for all countries within a species' distribution.

Charisma related variables were compiled from multiple sources. Maximum body size was sourced from Dulvy *et al.* [21], while body shape, pattern, achromatic contrast, chromatic contrast and colour uniqueness were all extracted from species illustrations in *Rays of the World* [29]. *Rays of the World* contains standardized illustrations and descriptions for 633 species of ray. Where taxonomies differed, we followed IUCN nomenclature and mapped data accordingly. Body shape was classified into five categories: disc, spade, diamond, wedge or rhombus. When body shape category was ambiguous based on the image alone, we defaulted to the shape description given in the species' *Identification* entry in *Rays of the World*. Patterning was also categorized into five categories: none, lined, spotted, mottled and other (where ‘other’ captured species patterns outside the main four types). In cases where multiple patterns were present the dominant one was recorded. Achromatic contrast, chromatic contrast and colour uniqueness were all quantified by analysing the standardized illustrations in *Rays of the World* [29]. We digitized illustrations and segmented them (removing all background elements) using the Grounded-SAM model from Idea Research [30], a development of the Segment Anything Model from Meta AI's Fundamental AI Research Lab [31]. Colour metrics were calculated using the R packages *recolorize* and *colordistance* [32,33]. All colour analysis was done within the CIELAB colour space, a colour space defined by the International Commission on Illumination (CIE) that represents colour using lightness (L) and two chromatic axes, green-red (a) and blue-yellow (b). CIELAB aims to achieve perceptual uniformity and thus maximize the representation of the human perception of colour differences.

We first re-clustered colours using the histogram methodology to reduce complexity and facilitate quantification, with a maximum of 10 clusters per colour channel. Clusters were subsequently merged if they differed by less than a specified threshold value. Several thresholds were trialled, aiming to reduce complexity while maintaining a minimum of two colours for any given species. The final threshold value was set to 12 and used across all species to ensure standardization of the approach.

We then calculated a weighted achromatic and chromatic colour contrast (figure 2) for all species, with weighting based on the proportional contribution of colours to the overall colouration of the animal, using the following formula:

$$\begin{array}{r} Contrast=\underset{i}{\sum }\underset{j}{\sum }w_{i}w_{j}\delta_{ij,} \end{array}$$

where i and j represent colour combinations present for a given species, $w_{i}$ and $w_{j}$ are the proportion of each species made up of colours i and j, and where $\delta_{ij}$ is the contrast between colours i and j. When i and j are the same colour $\delta_{ij}=\;0$. Colour uniqueness was derived from a pairwise colour distance matrix, followed by non-metric multidimensional scaling (NMDS, 3 dimensions, stress = 0.0349). Uniqueness was quantified as the Euclidean distance of each species from the multivariate centroid of all species (figure 2).

**Figure 2.**
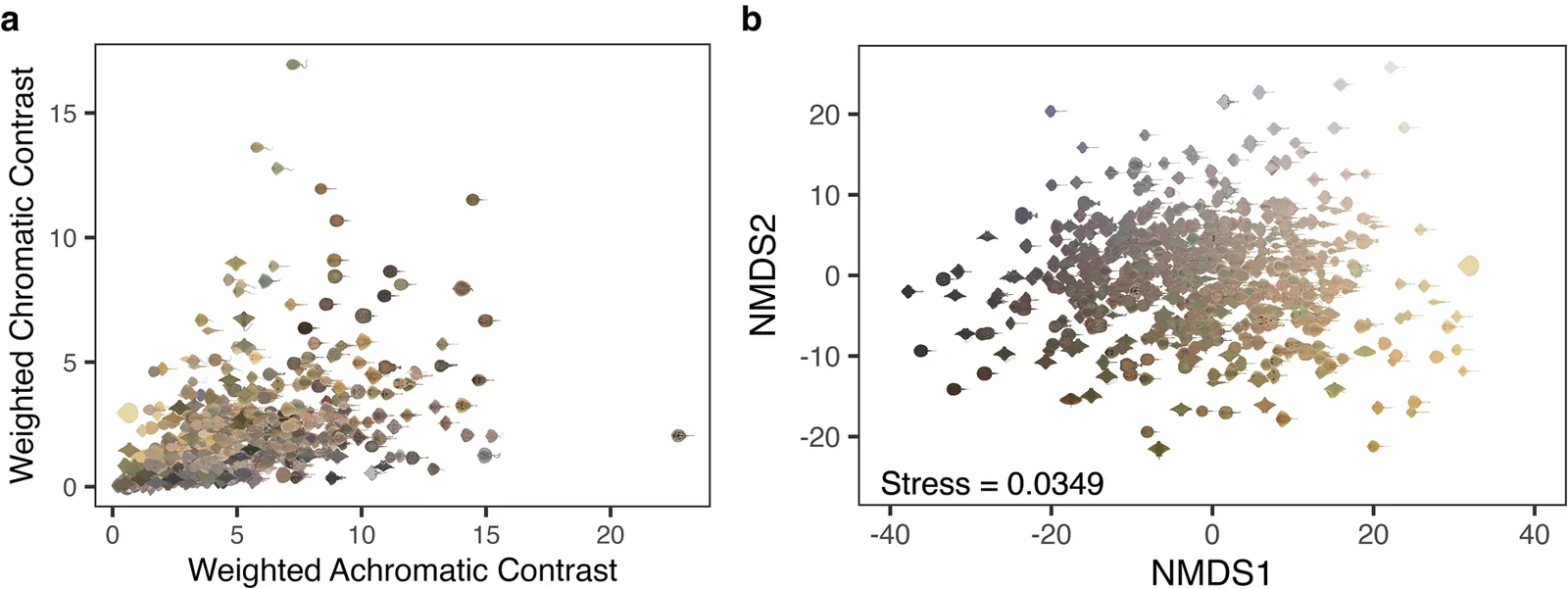
Distribution of ray (superorder Batoidea) colouration across 633 species. (a) Weighted achromatic and chromatic contrast of species. (b) Relative colour uniqueness of species based on the first two dimensions of a Non-Metric Multidimensional Scaling (NMDS) analysis of the hierarchical structure of species' colour. Original illustration by Dr. Lindsay Marshall, Stick Figure Fish Illustration. First published in *Rays of the World* (CSIRO Publishing, 2016). © 2025 Dr. Lindsay Marshall. Reused with acknowledgement to CSIRO Publishing. Further reuse requires permission from the copyright holder.

Finally, we collected data on research outputs for all 651 species through a literature search in Scopus (www.scopus.com), conducted on the 03 July 2025. We created a Boolean search term combining all current taxonomic names of species and their synonyms (see supplementary material). Searches were conducted for article titles, abstracts and keywords only and covered years from 1885 to 2025. The search returns (*n* = 7359) were filtered to include only finalized publications (i.e. those not *in press*) and constrained to articles, reviews, books, book chapters, data papers, letters and notes. A final list of 7124 papers was taken forward. Each paper was assigned to one or more species based on the presence of their taxonomic name or synonyms.

### (c) Analysis

Through the explicit encoding of causal links among variables, DAGs also imply independencies and/or conditional independencies among variables. For example, in our DAG (figure 1), the depth at which a species occurs is assumed to be conditionally independent (*d-separated*) from the total GDP across its geographic range after we control for the effect of geographic range. We need to first verify that these conditional independencies hold for our data [14]. Using the R package *dagitty* [34], we identified 35 conditional independencies in our DAG. We evaluated these conditional independencies (see supplementary material), using linear models for numeric and multinomial models for categorical variables, using base R and the package *nnet* [35]. We set *a priori* conditions for failure, requiring a *p*-value < 0.05 and an *R*^2^ > 0.05 to indicate a non-trivial proportion of variance explained. Bonferroni corrections for *p*-values were used to account for multiple hypothesis testing. All 35 conditional independencies were verified, supporting DAG-data consistency.

We then applied the backdoor criterion [14] to identify the appropriate sets of confounding variables, that is, those believed to be causal for both the response and variable of interest. When assessing the effect of one variable on research output, additional variables meet the backdoor criterion if the variable is not a descendant of the variable of interest (i.e. does not fall downstream along the causal pathway between the variable of interest and research output), and the variable closes a causal path that starts with a causal effect on the variable of interest. This procedure also serves to ensure we do not include collider variables (i.e. those that are caused by both the response and variable of interest) or overcontrol variables (i.e. those that are caused by the variable of interest, or those outside of the causal pathway) in our models. The final model specifications to estimate the causal effect of each variable of interest are presented in table 1. For each variable of interest, we estimate its average (marginal) causal effect on research output across all species. We do not fit interactions among predictors. Interaction effects are a distinct question from the confounding that the adjustment sets address, and their ommission does not bias these average effects.

**Table 1.** Model formulas for estimating the causal effect of each variable of interest plus their identified confounders, based on the application of the backdoor criterion to the directed acyclic graph representing the proposed causal structure of variables driving research output in rays (superorder Batoidea). Other than the taxonomy model, all models include taxonomy as a random effect error structure to account for non-independence among species.

variable	model formula for causal effect estimation
body shape	***research output ∼ body shape*** *+ taxonomy*
colour contrast	***research output ∼ colour contrast*** *+ body shape + depth + patterning + taxonomy*
colour uniqueness	***research output ∼ colour uniqueness*** *+ colour contrast + depth + patterning + taxonomy*
conservation status	***research output ∼ conservation status*** *+ depth + geographic range + gross domestic product + maximum body size + year of description + taxonomy*
depth	***research output ∼ depth*** *+ body shape + maximum body size + taxonomy*
geographic range	***research output ∼ geographic range*** *+ body shape + depth + maximum body size + taxonomy*
gross domestic product	***research output ∼ gross domestic product*** *+ geographic range + taxonomy*
patterning	***research output ∼ patterning*** *+ body shape + colour contrast + depth + taxonomy*
maximum body size	***research output ∼ maximum body size*** *+ body shape + taxonomy*
taxonomy	***research output ∼ taxonomy***
year of description	***research output ∼ year of description*** *+ depth + geographic range + gross domestic product + maximum body size + taxonomy*

Research output was log1p-transformed to reduce the influence of extreme outliers, and modelled using Bayesian mixed effect models with Gaussian assumptions and a random effect structure to account for taxonomy, except when taxonomy was itself the variable of interest, in which case it was instead included as a fixed effect. Models used four chains, with 4000 warmup iterations and 2000 sampling iterations. Weakly informative priors were specified for all models: a normal prior centred on the observed median log1p-transformed research output (*μ* = 1.1, *σ* = 1.5) was used for the intercept, providing a plausible baseline while permitting substantial uncertainty across the observed range of research effort; Student-*t* priors (df = 3, *μ* = 0, *σ* = 1) were used for all coefficients, providing mild regularization towards zero while accommodating genuine large effects through heavier tails than a normal prior; and a half-normal prior (*μ* = 0, *σ* = 1.5) was used for the residual standard deviation, consistent with the observed outcome standard deviation (s.d. = 1.33) while remaining diffuse over plausible values. Geographic range, minimum depth, maximum depth, GDP, body size, chromatic contrast and achromatic contrast were all log10-transformed before analysis to improve interpretability and reduce the potential influence of extreme values. Before modelling, numeric variables were scaled using Gelman's 2SD scaling, subtracting the median and dividing by two times the median absolute deviation. This scaling was chosen to facilitate approximate comparability between standardized effects for continuous and categorical variable comparison. Where relevant, pairwise contrasts were made using model draws. Finally, we constructed a standard correlative analysis for IUCN Red List Status and research output, to facilitate a comparison between our causal model outputs and more conventional approaches.

To assess the robustness of our causal estimates to unmeasured confounding, we computed *E-values* using the R package *EValue* [36] for each primary causal effect. The *E-value* quantifies the minimum strength of association, on the risk ratio scale, that an unmeasured confounder would need to have with both the exposure and the outcome, conditional on measured covariates, to fully explain away an observed effect. Model fit was assessed via posterior predictive checks and Bayesian *R*², though we note that individual causal models are not optimized to maximize model fit or predictive power. The goal of DAG-based causal inference is to estimate the causal effect of each variable while conditioning on the minimal sufficient adjustment set to block confounding paths, not to partition variance among predictors. *R*² should therefore be interpreted as a descriptive summary of explained variance rather than a measure of causal adequacy. Model fit metrics are available (see supplementary material).

All analyses were conducted using R v4.3.0 within RStudio [37,38]. For Bayesian models, we interpret effects where the 95% credible interval (CI) excludes 0 to be strong evidence of effect, where the 50% CI excludes 0 to be weak evidence, and where the 50% CI includes 0 to be no evidence of effect.

## 3. Results

Conditional on our assumed causal structure, our models reveal substantial and systemic biases in research outputs across the rays, with accessibility, charisma and conservation status each exerting strong influences on which species receive the most scientific attention.

### (a) Taxonomic bias

Research effort shows strong evidence for variability among taxonomic groupings, with Torpediniformes (*β* = 0.389, 95% CI (0.050, 0.729)), Myliobatiformes (*β* = 0.880, 95% CI (0.660, 1.10)) and Rhinopristiformes (*β* = 0.934, 95% CI (0.597, 1.263)) typically having greater research output that Rajiformes, and wide variability in research output at the family level (figure 3). At the family level species from the Pristidae (sawfishes) and Mobulidae (devil rays) typically had the highest levels of research output, and the Gurgesiellidae (pygmy skates) and Anacanthobatidae (smooth skates) had the least. The taxonomic model estimates per-species differences in research output among taxonomic groups. Effect sizes therefore reflect per-species bias, not aggregate differences driven by clade size. Subsequent exploration of accessibility, charisma and conservation status's causal effects on research biases allow us to begin to unpick why we see these taxonomic trends in research.

**Figure 3.**
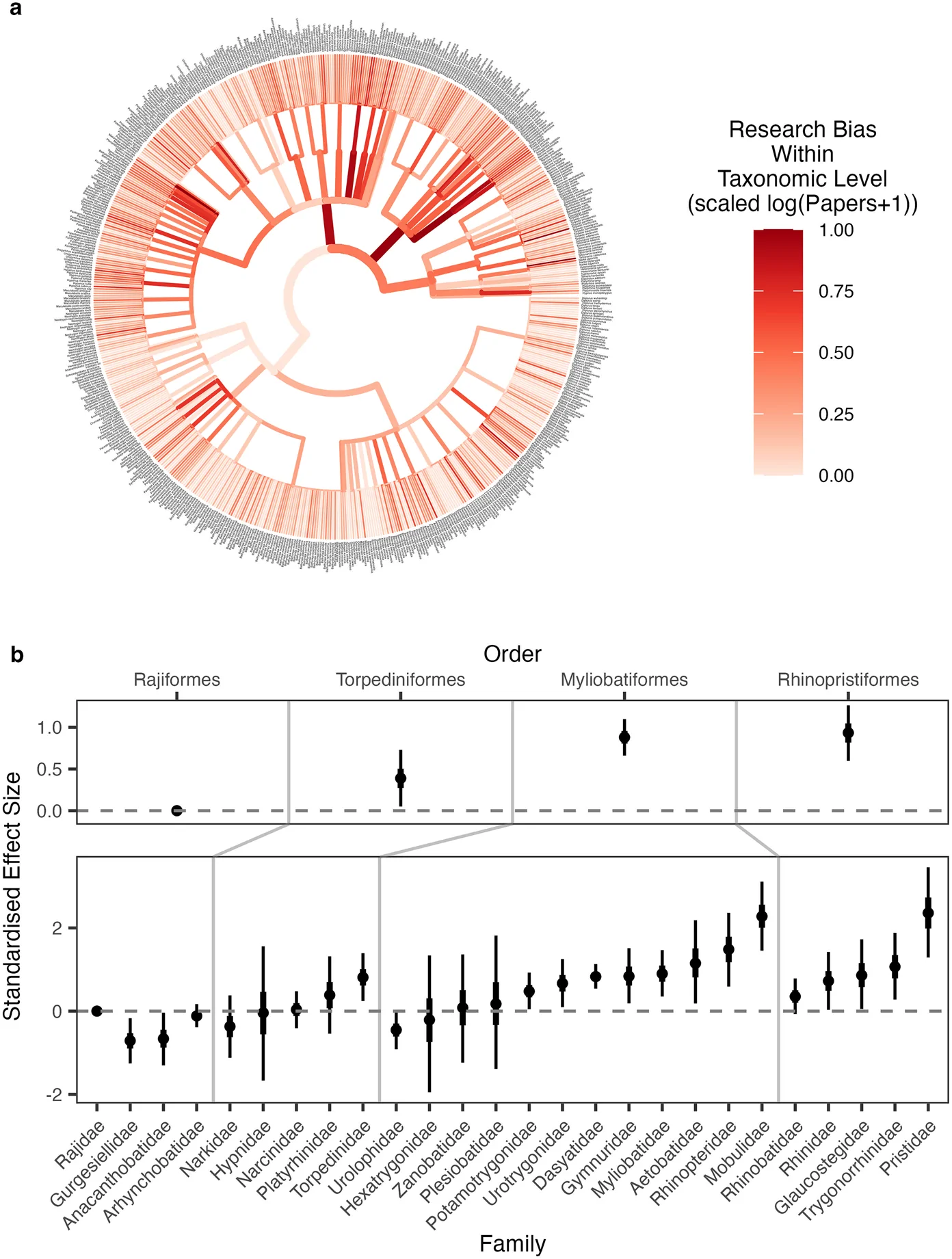
Taxonomic distribution and modelled bias in research output in rays and skates (superorder Batoidea). (a) Distribution of research papers identified in this study by taxonomy. (b) Bias in research output by order and family. Effect sizes represent posterior median *β* coefficients with 95% and 50% credible intervals on the log1p scale. Coefficients reflect differences relative to the reference category. Reference level for order is Rajiformes, reference level for family is Rajidae.

### (b) Accessibility biases

In terms of accessibility, there was strong evidence for research output being causally driven by inhabiting shallow minimum depths (*β* = −2.364, 95% CI (−2.778, −1.946)), earlier species description (*β* = −1.155, 95% CI (−1.369, −0.950)), broader geographic ranges (*β* = 0.581, 95% CI (0.425, 0.740)) and being distributed across an area with greater gross domestic product (*β* = 0.410, 95% CI (0.231, 0.589); figure 4). Interestingly, strong evidence was also found for increased research output with greater maximum depth (*β* = 0.600, 95% CI (0.246, 0.973)), suggesting that species spanning a wider vertical habitat range attract disproportionate research interest (figure 4). Among accessibility variables, minimum depth had the largest standardized effect on research outputs, followed by year of description (figure 4). The causal effect of minimum depth was the largest of all variables in our analysis.

**Figure 4.**
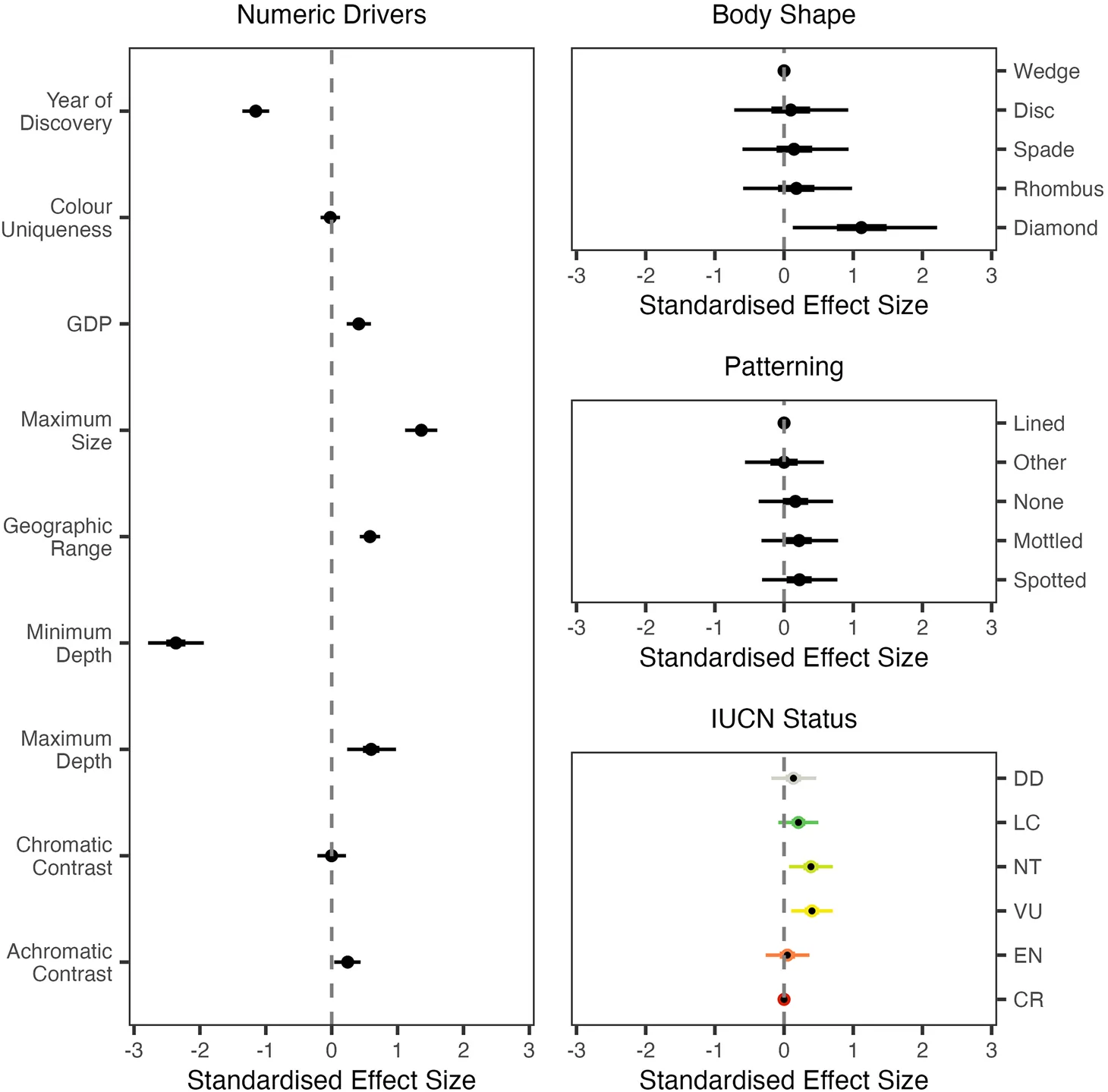
Standardized effect sizes for causal drivers of research output in rays and skates (superorder Batoidea) related to accessibility, charisma and endangered status. Effect sizes represent posterior median *β* coefficients with 95% and 50% credible intervals on the log1p scale. Coefficients for continuous predictors reflect the effect of a 2SD change in the predictor; coefficients for categorical predictors reflect differences relative to the reference category. Reference category for body shape is wedge, reference category for patterning is lined. Reference category for IUCN status is Critically Endangered.

### (c) Charisma bias

Charisma related traits also showed strong evidence for causal influence on research output. Larger-bodied species had greater research outputs (*β* = 1.359, 95% CI (1.112, 1.602)), as did species with stronger achromatic contrast (*β* = 0.243, 95% CI (0.042, 0.439)), and those species with diamond-shaped bodies had higher research output than species with wedge-shaped bodies (*β* = 1.126, 95% CI (0.115, 2.210); figure 4). There was also weak evidence to suggest that species with mottled (*β* = 0.218, 50% CI (0.030, 0.408)) and spotted (*β* = 0.227, 50% CI (0.044, 0.417)) patterning may have higher research output than those with lined patterns. However, neither chromatic contrast nor colour uniqueness showed any evidence of a causal effect on research output. Among charisma traits, body size had the largest causal effect and the second largest effect of any variable in our analysis (figure 4).

### (d) IUCN status bias

Finally, we found evidence for biases in research output driven by IUCN Red List Status. Concerningly, Critically Endangered species showed strong evidence for reduced research outputs compared to Vulnerable (*β* = 0.402, 95% CI (0.109, 0.699)) and Near Threatened species (*β* = 0.388, 95% CI (0.083, 0.699)), and weak evidence for reduced research compared to Least Concern (*β* = 0.209, 50% CI (0.110, 0.309)) and Data Deficient species (*β* = 0.138, 50% CI (0.024, 0.248)). Similarly, Endangered species showed strong evidence for reduced research outputs compared to Vulnerable (*β* = 0.355, 95% CI (0.083, 0.620)) and Near Threatened species (*β* = 0.340, 95% CI (0.053, 0.621)), and weak evidence for reduced research compared to Least Concern species (*β* = 0.161, 50% CI (0.070, 0.250)). Conversely, there was some weak evidence of Near Threatened species receiving greater research outputs than Data Deficient (*β* = −0.254, 50% CI (−0.346, −0.157)) and Least Concern species (*β* = −0.182, 50% CI (−0.263, −0.099)), and of Vulnerable species receiving greater research outputs than Data Deficient (*β* = −0.265, 50% CI (−0.358, −0.172)) and Least Concern species (*β* = −0.193, 50% CI (−0.273, −0.116)).

### (e) Correlative versus causally informed models of IUCN status impact

Comparing causal and correlative models of IUCN status effects emphasizes the value of causal inference. We found substantial differences between the naïve model and the causal model, most notably the naïve model finds that Critically Endangered species receive comparable research effort to all other conservation status levels, except Data Deficient species, which it determines receive less (*β* = −0.647, 95% CI (−1.066, −0.235)). It similarly suggests strong evidence that Data Deficient species receive less research than Endangered (*β* = −0.649, 95% CI (−1.037, −0.249)), Vulnerable (*β* = −0.881, 95% CI (−1.214, −0.557)), Near Threatened (*β* = −0.686, 95% CI (−1.039, −0.322)) and Least Concern species (*β* = −0.415, 95% CI (−0.701, −0.121)). This comparison demonstrates how correlative models can mischaracterize underlying causal relationships among variables by failing to account for appropriate confounding variables, while structured causal modelling provides a clearer view of the underlying causal drivers of research outputs.

### (f) Sensitivity analysis

To assess the robustness of our causal estimates to unmeasured confounding, we computed *E-*values [36] for all primary causal effects. The *E-value* quantifies the minimum strength of association, on the risk ratio scale, that an unmeasured confounder would need to have with both the exposure and the outcome, conditional on measured covariates, to fully explain away an observed effect. A risk ratio of 2.0, for example, indicates that an unmeasured confounder would need to at least double the risk of both the exposure and the outcome independently to nullify the observed effect. Larger *E-*values indicate greater robustness to unmeasured confounders. Our DAG already conditions on the known and hypothesized confounders for each estimand; *E-values* therefore represent the strength required of additional, unidentified confounders beyond those already modelled. For the two largest effects in our analysis, minimum depth (*E* = 18.04) and body size (*E* = 6.14), an unmeasured confounder would need to be associated with both the predictor and research output by a risk ratio exceeding 18-fold and 6-fold, respectively, well beyond what is plausible for any single unmeasured variable in this system. Year of description (*E* = 6.32), geographic range (*E* = 2.94), GDP (*E* = 2.29) and maximum depth (*E* = 2.93) were similarly robust. For IUCN Red List Status, *E-*values for the contrasts between Critically Endangered species and Vulnerable (*E* = 2.45) and Near Threatened species (*E* = 2.42) were greater than that of GDP, an uncontroversial driver of research effort, and are substantive given the breadth of confounders already accounted for in the model.

## 4. Discussion

Our causal analysis reveals substantial biases in ray research, conditional on the causal structure encoded in our DAG. Research outputs were driven by increases in the accessibility and charisma of species but away from those which are most threatened. We show that Critically Endangered and Endangered species receive levels of attention lower than Near Threatened and Vulnerable species and comparable to Data Deficient species, unless they happen to be highly accessible and/or charismatic. The lower research output for the most highly threatened species suggests the existence of a ‘Goldilocks’ zone, where species are threatened enough to justify research but not so threatened that research becomes logistically or practically prohibitive. Regardless, our results show that as a research community, we are not prioritizing the species most in need. This aversion may stem from systemic factors (e.g. funding biases, publication barriers and career incentives) as well as personal motivations (e.g. personal passions, drive for visibility or perceived risk of research failure) (e.g. [6,8,39]). Against the backdrop of the biodiversity crisis [1], neglecting the most threatened species only compounds their risk of extinction and diminishes our capacity to reverse biodiversity loss.

Instead, research is concentrated on species that are widely accessible and charismatic. Key traits driving higher research outputs include availability in shallow water depth, presence in deep water, broad geographic ranges, large body sizes, occurrence in wealthier regions and earlier year of description. Most of these relationships are unsurprising, reflecting lower research costs and greater ease of access. The exception is the seemingly paradoxical finding that research effort increases with both shallow minimum and deep maximum depths. However, this appears to be largely driven by two groups: oceanic deep-divers (e.g. *Pteroplatytrygon violacea*, *Mobula mobular*, *M. alfredi*, *M. birostris*), and exploited deepwater skates (e.g. *Dipturus chilensis*, *Leucoraja ocellata*, *Rostroraja eglanteria*, *Amblyraja radiata*, *Raja clavata*) that also inhabit shallow coastal zones. Most deep-sea obligates receive very little research attention, 88% of the 136 species with minimum depths of more than 200 m having one or fewer papers identified in this study. Research biases towards larger species are consistent with the wider literature. Larger body size is correlated with increased perceptions of charisma and ecological importance, as well as to greater commercial value [13,40,41]. These perceptions in turn attract wider public interest and funding [8,13]. Increasing wealth across a species' range also positively influences research, probably through greater access to research funding and infrastructure [42]. However, the scale of the wealth effect was smaller than might have been anticipated given the strong GDP–research capacity relationships documented at the country level. Several mechanisms probably attenuate this relationship: international conservation aid to lower-income countries can approach domestic conservation spending in scale [43], and international research collaborations may further decouple research activity from local economic conditions [44]. Nevertheless, international funding flows are themselves taxonomically biased [8], and the disparity in research capacity between high- and low-capacity nations continues to grow [44], suggesting these compensatory mechanisms remain insufficient to close the wealth-driven research gap.

Consistent with findings in other taxa [7,18,19], we also found research biases linked to species' morphology and colouration, reflecting the impacts of perceived charisma. Notably, research outputs were highest in species with diamond-shaped discs (aquilloformes like mobulid and eagle rays), the rarest disc type, suggesting uniqueness of form may play an important role in perceived charisma. Relative visual uniqueness is a known driver of human preference in non-human objects [45]. The shark-like body shape of Rhinopristiformes, while distinctive among rays, is similar to that of most shark species, potentially diminishing its perceived uniqueness and so explaining the lower research output associated with this body shape. Surprisingly, while we found strong evidence for an effect of achromatic contrast, its effect size was small, and there was no evidence of bias with chromatic contrast or colour uniqueness, despite traits associated with colour uniqueness influencing perceptions in other taxa like bony fishes, snakes and birds [7,19,46,47]. Rays display relatively little colour variability compared to these taxa, most species sit on a continuum of blacks, greys, browns and beiges, which may reduce the influence of colour-based traits on research patterns. Additionally, colour and patterning traits in batoids tend to be strongly phylogenetically conserved, making these effects difficult to isolate from those already captured by taxonomy. Body size and shape, while also phylogenetically structured, show greater within-family and within-order variation, potentially explaining their stronger detectable effects.

While biases in research with accessibility and charisma are to be expected, when coupled with an aversion to working on the most threatened species they present a significant barrier to effective conservation. The potential consequences of these research biases are starkly illustrated by the Java stingaree (*Urolophus javanicus*), the first documented extinction of a marine fish owing to human activities [23]. Despite being described as early as 1864 and occurring in shallow waters, this species received very little research attention before its extinction. The species combines low charisma traits (small, disc-shaped, non-patterned) with a restricted geographic range in a low-income region and had been listed as Critically Endangered since 2006, driving low levels of research interest. The Java stingaree highlights how systemic biases in research can directly undermine conservation prospects. It is unlikely that this will be the last ray to go extinct; numerous high-risk species with similar life histories and negligible research attention remain (table 2). To have a chance of saving them we must tackle systematic research biases urgently.

**Table 2.** Priority species for research—Critically Endangered and Endangered species with three or fewer peer-reviewed papers identified in this study.

**critically endangered species**		
*Acroteriobatus variegatus*	*Glaucostegus younholeei*	*Rhinobatos lionotus*
*Dentiraja confusa*	*Gymnura tentaculata*	*Rhinobatos whitei*
*Dipturus mennii*	*Hongeo koreana*	*Rhynchobatus immaculatus*
*Fontitrygon colarensis*	*Hypanus rudis*	*Rhynchobatus luebberti*
*Fontitrygon garouaensis*	*Leucoraja melitensis*	*Rhynchobatus mononoke*
*Fontitrygon geijskesi*	*Maculabatis bineeshi*	*Rhynchobatus springeri*
*Fontitrygon ukpam*	*Pastinachus stellurostris*	*Rhynchorhina mauritaniensis*
*Glaucostegus obtusus*	*Rhinobatos albomaculatus*	*Torpedo suessii*
*Glaucostegus thouin*	*Rhinobatos irvinei*	*Urotrygon reticulata*
**endangered species**		
*Acroteriobatus leucospilus*	*Hemitrygon sinensis*	*Rhinobatos borneensis*
*Aetomylaeus milvus*	*Himantura undulata*	*Telatrygon crozieri*
*Brevitrygon javaensis*	*Maculabatis pastinacoides*	*Torpedo adenensis*
*Dipturus canutus*	*Narcine prodorsalis*	*Torpedo bauchotae*
*Dipturus maugeanus*	*Pastinachus gracilicaudus*	*Torpedo mackayana*
*Fluvitrygon kittipongi*	*Pateobatis bleekeri*	*Torpedo panthera*
*Fluvitrygon oxyrhynchus*	*Potamotrygon tigrina*	*Urogymnus lobistoma*
*Gymnura sereti*	*Raja ocellifera*	*Urolophus orarius*

Overcoming biases in research to realign them with conservation priorities will require actively challenging the *status quo*. There is no simple solution to this issue, but there are several steps we could take that may help. First, our findings could directly inform the design of funding calls by providing an evidence-based framework for redirecting research towards neglected taxa. While funding initiatives such as those from the Save Our Seas Foundation and Shark Conservation Fund have undoubtedly advanced work on high-profile threatened taxa (e.g. sawfishes, mobulids, wedgefishes), support for equally threatened but less charismatic species remains minimal. Expanding or redirecting funding towards species that are threatened but score low on the biological and socioeconomic drivers of research effort identified here (e.g. small ranges, deep water, low charisma, low GDP range states) will be critical to building an evidence base to support effective conservation. Second, publication incentives could help to rebalance research, for example through special issues on neglected taxa in flagship journals, and through proactive solicitation and peer-review of the many existing but unpublished datasets held by researchers, government agencies or NGOs [48]. Third, conservation marketing has a vital role: public and policymaker attention is often skewed towards perceived charisma [49,50]. Targeted campaigns that frame overlooked rays as ecologically vital, where this is scientifically defensible, or ‘quirky’ yet imperilled could help to mobilize both funding and political will. Finally, greater integration and promotion of under-researched species within international policy frameworks, such as the Convention on International Trade in Endangered Species (CITES) or the Conservation of Migratory Species (CMS), and ongoing international programmess like the Important Shark and Ray Areas (ISRA) [51] could help to formalize their conservation importance, generate policy leverage and direct resources towards them. However, realizing this latter goal faces significant barriers: listing processes typically require baseline population data that are unavailable for data-deficient species, and political will among range states is often insufficient for species with low economic or cultural visibility.

This study identifies important causal drivers of research bias in rays, but the estimates are, as with all causal inference from observational data, conditional on the assumed causal structure. A key advantage of the DAG framework is that we make these assumptions transparent and testable, rather than embedded implicitly in model specification decisions. Two particular assumptions in our study warrant emphasis. First, the DAG encodes contemporaneous, unidirectional relationships and cannot represent dynamic feedback; in particular, we model conservation status as a cause of research output. A bidirectional pathway is conceivable, whereby greater research generates data that informs conservation efforts and produces improved and/or better informed subsequent IUCN reassessments. However, at present only four (two of which are batoids) of the approximately 1200 shark and ray species have shown signs of improved conservation status [21], providing little signal from which a feedback loop could be estimated with cross-sectional data. Second, our estimates are average causal effects across species; we did not model effect modification (variation in a variable's effect across the levels of another), which is a distinct question from confounding and does not bias the averages reported here. Exploration of both of these elements, where or when data allow, would be valuable in further understanding bias in batoid research and the benefits of heightened research effort for the most endangered species. We encourage future studies to build on the transparency of our approach here, by proposing and testing alternative causal structures wherever domain knowledge supports them. Extending causal analyses to other taxonomic groups will help determine whether these dynamics are generalized or taxon-specific. Longitudinal analyses could clarify temporal shifts, particularly in response to corrective interventions such as targeted funding or conservation marketing, as well as evaluating potential lags between species threat listings and subsequent research outputs.

Our work examined the peer-reviewed literature, and so does not integrate additional knowledge held in grey literature, governmental databases or NGO archives. Incorporation of additional sources may alter (probably strengthening) measured biases and may help to better identify bottlenecks in the transfer of knowledge into the peer-reviewed literature. Direct exploration of patterns in research funding itself, which ultimately drives research output at a stage more fundamental than publication, would be valuable, though comprehensive, species-resolved funding databases for batoids are not currently available at the scale required for such analyses. Future works could also integrate cultural, economic and social dimensions, which may drive research efforts and are key to harness for conservation effectiveness [52]. These dimensions are often difficult to quantify, especially across multiple species: economic value in many cases is not recorded at the species level, cultural significance lacks universally applicable metrics and social values are often inherently context dependent. Developing systematic proxies for these values would allow their inclusion in causal frameworks and improve understanding of how they shape research priorities.

To conclude, our findings reveal a troubling reality: rays most in need of research attention are studied the least, a result of entrenched and multifaceted biases in conservation science. Recalibrating research priorities is both an ethical obligation and a strategic necessity in addressing the biodiversity crisis. Explicitly acknowledging these biases enables us to chart pathways towards a more equitable and effective conservation agenda. Causal modelling, as demonstrated here, provides a powerful framework for disentangling complex drivers of bias and guiding targeted, evidence-based strategies to align research effort with conservation need.

## Supplementary Material



## Data Availability

All code and data required to replicate the analyses in this article are available through Zenodo [53]. All 633 images of rays and skates used in this work are available in the species description sections of *Rays of the World* [29]. Supplementary material is available online [54].
