# Supplementary material for: Too threatened to try: how extinction risk drives research neglect in rays and skates
Source: Proc Biol Sci. 2026 Nov 4;293(2082):20262126. doi: 10.1098/rspb.2026.2126 (PMC13730809; doi:10.1098/rspb.2026.2126)
Supplement: Supplementary file 1 [file rspb.2026.2126.zip › rspb.2026.2126/rspb20262126_si_001.docx]

**Supplementary Materials**

***Andrew J. Temple, Sophia Rosinski, Jesse E.M. Cochran, Faizan F. Khan, Lindsay Marshall, Michael L. Berumen. Too threatened to try: how extinction risk drives research neglect in rays and skates. Proceedings of the Royal Society B. doi:10.1098/rspb.2026.2126.***

***Directed acyclic graph justifications***

Justifications for theorised causal links underlying the directed acyclic graph used in this study.

| **Parent Node** | **Child Node** | **Justification** |
| --- | --- | --- |
| Colour Contrast | Scientific Papers | Visually striking species with high chromatic or achromatic contrast may attract disproportionate research attention due to their aesthetic appeal and visibility to researchers and the public (Langlois et al., 2022). |
| Colour Contrast | Colour Uniqueness | Colour uniqueness contributes directly to the perceptual distinctiveness. High colour uniqueness may attract disproportionate research attention due to their aesthetic appeal and visibility. |
| Depth | Scientific Papers | Species distributed in shallow, accessible waters are more frequently encountered by researchers and are easier to sample, which would be expected to lead to higher research output. |
| Depth | Colour Phenotype | Light availability decreases rapidly with depth, imposing strong selective pressures on colour phenotype for crypsis and background matching in response to vision limitations in these environments (Lythgoe, 1979). |
| Depth | Geographic Range | Shallow coastal habitats are geographically fragmented, constraining species to smaller 2D ranges, whereas deeper and/or more pelagic species have access to continuous, extensive habitat. |
| Depth | Conservation Status | Shallow-water species face greater anthropogenic pressures including coastal habitat degradation, bycatch, and targeted fishing, increasing their likelihood of receiving a threatened IUCN status (Dulvy et al., 2024, 2021). |
| Depth | Colour Uniqueness | Depth-related constraints on colour phenotype (Lythgoe, 1979) are expected to produce convergent colouration among deep-water species, reducing their colour uniqueness relative to shallow species with more diverse colour phenotypes. |
| Depth | Year of Description | Deep-water species are harder to sample and observe, resulting in later taxonomic discovery relative to shallow-water species that are more readily encountered (Costello et al., 2015). |
| Patterning | Scientific Papers | Distinctively patterned species may attract greater research attention due to their visual appeal to researchers and the public. |
| Patterning | Colour Uniqueness | Complex or distinctive colour patterns may increase the quantitative distinctiveness of a species relative to others in the same assemblage because their existence requires contrasting colours. |
| Colour Phenotype | Colour Contrast | The underlying colour phenotype of a species directly determines the measurable chromatic and achromatic contrast of the dorsal surface. |
| Colour Phenotype | Patterning | The spatial arrangement of colour elements is a direct expression of the underlying colour phenotype. |
| Geographic Range | Scientific Papers | Wide-ranging species are encountered by more researchers across more nations, increasing the probability of research attention and publication. |
| Geographic Range | Conservation Status | Restricted geographic range is an explicit criterion for threatened status under IUCN Red List categories, and small-ranged species face greater extinction risk from localised threats. |
| Geographic Range | Gross Domestic Product | Species with larger geographic ranges overlap with more nations and therefore accumulate higher cumulative GDP across their range states. |
| Geographic Range | Year of Description | Wide-ranging species are more likely to be encountered, leading to earlier taxonomic description (Costello et al., 2015; Moura and Jetz, 2021). |
| Body Shape | Depth | Body shape in batoids reflects ecological guild and habitat affinity, with benthic disc-shaped species often associated with shallow coastal habitats whereas spade- or diamond-shaped species are associated with deeper or more pelagic environments. |
| Body Shape | Scientific Papers | Body shape may influence human perception of charisma and scientific interest, with morphologically distinctive species potentially attracting disproportionate research attention independently of other biological traits. |
| Body Shape | Colour Phenotype | Body shape reflects ecological guild and habitat affinity, both of which are expected to determine the crypsis requirements and/or background-matching strategies that drive colour phenotype. |
| Body Shape | Geographic Range | Body shape proxy’s ecological specialisation, with specialist body plans such as those of aquillomorphs (diamond shaped) facilitating wider ranging and/or pelagic movements. |
| Body Shape | Maximum Body Size | Body plan reflects ecological guild and lifestyle which may constrain the maximum attainable body size. |
| Maximum Body Size | Depth | Larger fish species tend to be depth generalists with broader depth ranges but may be inverse in pelagic elasmobranchs (Smith and Brown, 2002). |
| Maximum Body Size | Scientific Papers | Larger species are more conspicuous, more charismatic, more likely to interact with humans. They are disproportionately targeted by both fisheries and conservation research, leading to higher research output (e.g. Clark and May, 2002; Titley et al., 2017). |
| Maximum Body Size | Geographic Range | Larger fish species tend to have larger geographic ranges, likely a result of dispersal capacity (Luiz et al., 2013; Strona et al., 2012). |
| Maximum Body Size | Conservation Status | Larger species have slower life histories including later maturity, lower reproductive rates, and longer lifespans, making them more vulnerable to overexploitation and increasing their likelihood of receiving a threatened IUCN status (Dulvy et al., 2021; Reynolds et al., 2005). |
| Maximum Body Size | Year of Description | Larger, more conspicuous species are more likely to be discovered and described early (Costello et al., 2015; Moura and Jetz, 2021). |
| Conservation Status | Scientific Papers | Threatened species should attract conservation-motivated research and IUCN listing may increases research attention by signalling conservation need to the scientific community and funding bodies. Evidence is mixed (e.g. de Oliveira et al., 2023; Dos Santos et al., 2020; Guedes et al., 2023; Guénard et al., 2025; Mammola et al., 2023; Titley et al., 2017). |
| Gross Domestic Product | Scientific Papers | Nations with higher GDP have greater capacity to fund and conduct scientific research. |
| Gross Domestic Product | Conservation Status | Wealthier nations often have greater fishing capacity and more developed fisheries industries, but also greater capacity to effectively managed biodiversity (Melnychuk et al., 2017). |
| Gross Domestic Product | Year of Description | Wealthier nations have historically had greater capacity for natural history exploration and taxonomic research, species within their waters are often described earlier (Costello et al., 2015). |
| Colour Uniqueness | Scientific Papers | Colour-unique species are more visually distinctive which may attract greater research interest if this perceived novelty translates to increased aesthetic appeal (Langlois et al., 2022). |
| Year of Description | Scientific Papers | Species described earlier have had more time to accumulate research output. |
| Year of Description | Conservation Status | Recently described species are more likely to be assessed as Data Deficient by the IUCN due to insufficient population trend data, life history information, and distributional records needed for a full assessment (Bland et al., 2017). |

***Conditional Independence Tests***

Conditional independence tests for d-separated variables in the Directed Acyclic Graph. *a priori* conditions for failure: adjusted *p*-value < 0.05 and a non-trivial proportion of variance explained in the response *R^2^* > 0.05 (5% of variance explained in the response). Variables separated by _||_ are d-separated. Variables appearing after | are those that d-separation is dependent on.

| **Model** | **Model type** | ***p*-value** | ***p*-value adjusted** | **Partial *R^2^*** | **d-separation failure?** |
| --- | --- | --- | --- | --- | --- |
| Colour Contrast (Chromatic) _\|\|_ Geographic Range \| Depth, Body Body Shape | Linear model | 0.729 | 1.000 | <0.01 | No |
| Colour Contrast (Achromatic) _\|\|_ Geographic Range \| Depth, Body Body Shape | Linear model | 0.032 | 1.000 | 0.01 | No |
| Colour Contrast (Chromatic) _\|\|_ Maximum Body Size \| Depth, Body Shape | Linear model | 0.000 | 0.002 | 0.03 | No |
| Colour Contrast (Achromatic) _\|\|_ Maximum Body Size \| Depth, Body Shape | Linear model | 0.445 | 1.000 | <0.01 | No |
| Colour Contrast (Chromatic) _\|\|_ Conservation Status \| Depth, Geographic Range, Maximum Body Size | Linear model | 0.498 | 1.000 | 0.01 | No |
| Colour Contrast (Achromatic) _\|\|_ Conservation Status \| Depth, Geographic Range, Maximum Body Size | Linear model | 0.164 | 1.000 | 0.01 | No |
| Colour Contrast (Chromatic) _\|\|_ Conservation Status \| Depth, Body Shape | Linear model | 0.250 | 1.000 | 0.01 | No |
| Colour Contrast (Achromatic) _\|\|_ Conservation Status \| Depth, Body Shape | Linear model | 0.107 | 1.000 | 0.02 | No |
| Colour Contrast (Chromatic) _\|\|_ Gross Domestic Product \| Geographic Range | Linear model | 0.024 | 0.827 | 0.01 | No |
| Colour Contrast (Achromatic) _\|\|_ Gross Domestic Product \| Geographic Range | Linear model | 0.474 | 1.000 | <0.01 | No |
| Colour Contrast (Chromatic) _\|\|_ Gross Domestic Product \| Depth, Body Shape | Linear model | 0.052 | 1.000 | 0.01 | No |
| Colour Contrast (Achromatic) _\|\|_ Gross Domestic Product \| Depth, Body Shape | Linear model | 0.249 | 1.000 | <0.01 | No |
| Colour Contrast (Chromatic) _\|\|_ Year of Description \| Depth, Geographic Range, Maximum Body Size | Linear model | 0.091 | 1.000 | <0.01 | No |
| Colour Contrast (Achromatic) _\|\|_ Year of Description \| Depth, Geographic Range, Maximum Body Size | Linear model | 0.140 | 1.000 | <0.01 | No |
| Depth (Minimum) _\|\|_ Gross Domestic Product \| Geographic Range | Linear model | 0.164 | 1.000 | 0.01 | No |
| Depth (Maximum) _\|\|_ Gross Domestic Product \| Geographic Range | Linear model | 0.449 | 1.000 | <0.01 | No |
| Patterning _\|\|_ Geographic Range \| Depth, Body Shape | Multinomial | 0.024 | 0.826 | NA | No |
| Patterning _\|\|_ Maximum Body Size \| Depth, Body Shape | Multinomial | 0.200 | 1.000 | NA | No |
| Patterning _\|\|_ Conservation Status \| Depth, Geographic Range, Maximum Body Size | Multinomial | 0.367 | 1.000 | NA | No |
| Patterning _\|\|_ Conservation Status \| Depth, Body Shape | Multinomial | 0.401 | 1.000 | NA | No |
| Patterning _\|\|_ Gross Domestic Product \| Geographic Range | Multinomial | 0.166 | 1.000 | NA | No |
| Patterning _\|\|_ Gross Domestic Product \| Depth, Body Shape | Multinomial | 0.095 | 1.000 | NA | No |
| Patterning _\|\|_ Year of Description \| Depth, Geographic Range, Maximum Body Size | Multinomial | 0.404 | 1.000 | NA | No |
| Patterning _\|\|_ Year of Description \| Depth, Body Shape | Multinomial | 0.544 | 1.000 | NA | No |
| Geographic Range _\|\|_ Colour Uniqueness \| Colour Contrast, Depth, Patterning | Linear model | 0.829 | 1.000 | <0.01 | No |
| Geographic Range _\|\|_ Colour Uniqueness \| Depth, Body Shape | Linear model | 0.703 | 1.000 | <0.01 | No |
| Body Shape _\|\|_ Conservation Status \| Depth, Geographic Range, Maximum Body Size | Multinomial | 0.410 | 1.000 | NA | No |
| Body Shape _\|\|_ Gross Domestic Product \| Geographic Range | Multinomial | 0.449 | 1.000 | NA | No |
| Body Shape _\|\|_ Colour Uniqueness \| Colour Contrast, Depth, Patterning | Multinomial | 0.119 | 1.000 | NA | No |
| Body Shape _\|\|_ Year of Description \| Depth, Geographic Range, Maximum Body Size | Multinomial | 0.992 | 1.000 | NA | No |
| Maximum Body Size _\|\|_ Gross Domestic Product \| Geographic Range | Linear model | 0.502 | 1.000 | <0.01 | No |
| Maximum Body Size _\|\|_ Colour Uniqueness \| Colour Contrast, Depth, Patterning | Linear model | 0.036 | 1.000 | <0.01 | No |
| Maximum Body Size _\|\|_ Colour Uniqueness \| Depth, Body Shape | Linear model | 0.456 | 1.000 | 0.03 | No |
| Conservation Status _\|\|_ Colour Uniqueness \| Colour Contrast, Depth, Patterning | Multinomial | 0.740 | 1.000 | NA | No |
| Conservation Status _\|\|_ Colour Uniqueness \| Depth, Body Shape | Multinomial | 0.000 | 0.000 | NA | No |
| Conservation Status _\|\|_ Colour Uniqueness \| Depth, Geographic Range, Maximum Body Size | Multinomial | 0.288 | 1.000 | NA | No |
| Gross Domestic Product _\|\|_ Colour Uniqueness \| Colour Contrast, Depth, Patterning | Linear model | 0.324 | 1.000 | <0.01 | No |
| Gross Domestic Product _\|\|_ Colour Uniqueness \| Depth, Body Shape | Linear model | 0.315 | 1.000 | <0.01 | No |
| Gross Domestic Product _\|\|_ Colour Uniqueness \| Geographic Range | Linear model | 0.400 | 1.000 | <0.01 | No |
| Colour Uniqueness _\|\|_ Year of Description \| Depth, Geographic Range, Maximum Body Size | Linear model | 0.423 | 1.000 | 0.01 | No |
| Colour Uniqueness _\|\|_ Year of Description \| Depth, Body Shape | Linear model | 0.496 | 1.000 | 0.01 | No |
| Colour Uniqueness _\|\|_ Year of Description \| Colour Contrast (), Depth, Patterning | Linear model | 0.025 | 0.888 | 0.01 | No |

***Causal Model Performance Metrics***

Bayesian *R^2^* metrics for causal models. Note that individual causal models are not optimised for predictive power and *R²* should be interpreted as a descriptive summary of explained variance rather than a measure of causal adequacy.

| **Causal variable of interest** | **Model** | **Bayesian R^2^** |
| --- | --- | --- |
| Body Shape | ***Research Output ~ Body Shape*** *+ Taxonomy* | 0.324 95% CI [0.259, 0.387] |
| Colour Contrast | ***Research Output ~ Colour Contrast*** *+ Body Shape + Depth + Patterning + Taxonomy* | 0.444 95% CI [0.390, 0.493] |
| Colour Uniqueness | ***Research Output ~ Colour Uniqueness*** *+ Colour Contrast + Depth + Patterning + Taxonomy* | 0.441 95% CI [0.387, 0.491] |
| Conservation Status | ***Research Output ~ Conservation Status*** *+ Depth + Geographic Range + Gross Domestic Product + Maximum Body Size + Year of Description + Taxonomy* | 0.601 95% CI [0.563, 0.635] |
| Depth | ***Research Output ~ Depth*** *+ Body Shape + Maximum Body Size + Taxonomy* | 0.478 95% CI [0.429, 0.522] |
| Geographic Range | ***Research Output ~ Geographic Range*** *+ Body Shape + Depth + Maximum Body Size + Taxonomy* | 0.520 95% CI [0.475, 0.561] |
| Gross Domestic Product | ***Research Output ~ Gross Domestic Product*** *+ Geographic Range + Taxonomy* | 0.456 95% CI [0.401, 0.505] |
| Patterning | ***Research Output ~ Patterning*** *+ Body Shape + Colour Contrast + Depth + Taxonomy* | 0.442 95% CI [0.388, 0.491] |
| Maximum Body Size | ***Research Output ~ Maximum Body Size*** *+ Body Shape + Taxonomy* | 0.411 95% CI [0.356, 0.465] |
| Taxonomy | ***Research Output ~ Taxonomy*** | Order: 0.105 95% CI [0.065, 0.148] Family: 0.206 95% CI [0.158, 0.253] |
| Year of Description | ***Research Output ~ Year of Description*** *+ Depth + Geographic Range + Gross Domestic Product + Maximum Body Size + Taxonomy* | 0.591 95% CI [0.552, 0.626] |

***Boolean search term***

Boolean search term used in the collation of literature from Scopus:

*TITLE-ABS-KEY("Acroteriobatus annulatus" OR "Rhinobatos annulatus" OR "Acroteriobatus blochii" OR "Rhinobatos blochii" OR "Acroteriobatus leucospilus" OR "Rhinobatos leucospilus" OR "Acroteriobatus ocellatus" OR "Rhinobatos ocellatus" OR "Acroteriobatus omanensis" OR "Acroteriobatus salalah" OR "Rhinobatos salalah" OR "Acroteriobatus variegatus" OR "Rhinobatos variegatus" OR "Acroteriobatus zanzibarensis" OR "Rhinobatos zanzibarensis" OR "Aetobatus flagellum" OR "Raja flagellum" OR "Aetobatus laticeps" OR "Aetobatis laticeps" OR "Aetobatus narinari" OR "Raja narinari" OR "Aetobatus narutobiei" OR "Aetobatus ocellatus" OR "Aetobatus guttatus" OR "Myliobatus ocellatus" OR "Aetomylaeus ocellatus" OR "Raja mula" OR "Raja guttata" OR "Myliobatis macroptera" OR "Aetobatus punctatus" OR "Myliobatis eeltenkee" OR "Aetomylaeus asperrimus" OR "Pteromylaeus asperrimus" OR "Myliobatis asperrimus" OR "Aetomylaeus bovinus" OR "Pteromylaeus bovina" OR "Pteromylaeus bovinus" OR "Myliobatis bovina" OR "Aetomylaeus caeruleofasciatus" OR "Aetomylaeus maculatus" OR "Myliobatus maculatus" OR "Aetomylaeus milvus" OR "Myliobatis milvus" OR "Aetomylaeus nichofii" OR "Raja niehofii" OR "Raja nichofii" OR "Aetomylus huletti" OR "Aetomylaeus vespertilio" OR "Aetomylaeus reticulates" OR "Myliobatis vespertilio" OR "Amblyraja badia" OR "Raja badia" OR "Amblyraja doellojuradoi" OR "Raja doellojuradoi" OR "Amblyraja frerichsi" OR "Raja frerichsi" OR "Amblyraja georgiana" OR "Raja georgiana" OR "Amblyraja hyperborea" OR "Raja hyperborea" OR "Amblyraja jenseni" OR "Raja jenseni" OR "Amblyraja radiata" OR "Raja radiata" OR "Raia scabrata" OR "Amblyraja reversa" OR "Raia reversa" OR "Amblyraja robertsi" OR "Raja robertsi" OR "Amblyraja taaf" OR "Raja taaf" OR "Anacanthobatis marmorata" OR "Leiobatis marmoratus" OR "Anacanthobatis marmoratus" OR "Anoxypristis cuspidata" OR "Pristis cuspidatus" OR "Aptychotrema rostrata" OR "Raja rostrata" OR "Aptychotrema timorensis" OR "Aptychotrema vincentiana" OR "Rhinobates vincentianus" OR "Arctoraja sexoculata" OR "Bathyraja sexoculata" OR "Arhynchobatis asperrimus" OR "Atlantoraja castelnaui" OR "Raja castelnaui" OR "Atlantoraja cyclophora" OR "Raia cyclophora" OR "Atlantoraja platana" OR "Raja platana" OR "Bathyraja abyssicola" OR "Raja abyssicola" OR "Bathyraja aguja" OR "Raia aguja" OR "Bathyraja albomaculata" OR "Rhinoraja albomaculata" OR "Raja albomaculata" OR "Bathyraja aleutica" OR "Raja aleutica" OR "Bathyraja andriashevi" OR "Bathyraja arctowskii" OR "Raja arctowskii" OR "Bathyraja bergi" OR "Bathyraja pseudoisotrachys" OR "Bathyraja brachyurops" OR "Raja brachyurops" OR "Bathyraja cousseauae" OR "Bathyraja diplotaenia" OR "Breviraja diplotaenia" OR "Rhinoraja diplotaenia" OR "Bathyraja eatonii" OR "Bathyraja fedorovi" OR "Rhinoraja fedorovi" OR "Bathyraja griseocauda" OR "Raja griseocauda" OR "Bathyraja hesperafricana" OR "Bathyraja interrupta" OR "Rhinoraja interrupta" OR "Raia interrupta" OR "Bathyraja irrasa" OR "Bathyraja ishiharai" OR "Bathyraja isotrachys" OR "Breviraja isotrachys" OR "Rhinoraja isotrachys" OR "Raja isotrachys" OR "Bathyraja kincaidii" OR "Raia kincaidii" OR "Bathyraja leucomelanos" OR "Bathyraja lindbergi" OR "Bathyraja longicauda" OR "Breviraja longicauda" OR "Bathyraja maccaini" OR "Bathyraja macloviana" OR "Raja macloviana" OR "Rhinoraja macloviana" OR "Bathyraja maculata" OR "Bathyraja magellanica" OR "Raja magellanica" OR "Rhinoraja magellanica" OR "Bathyraja mariposa" OR "Bathyraja matsubarai" OR "Bathyraja notoroensis" OR "Bathyraja caeluronigricans" OR "Breviraja matsubarai" OR "Bathyraja meridionalis" OR "Bathyraja microtrachys" OR "Bathyraja minispinosa" OR "Bathyraja multispinis" OR "Raja multispinis" OR "Rhinoraja multispinis" OR "Bathyraja murrayi" OR "Raja murrati" OR "Rhinoraja murrayi" OR "Bathyraja pacifica" OR "Bathyraja pallida" OR "Breviraja pallida" OR "Bathyraja panthera" OR "Bathyraja papilionifera" OR "Bathyraja papilonifera" OR "Bathyraja parmifera" OR "Arctoraja parmifera" OR "Raia parmifera" OR "Bathyraja peruana" OR "Bathyraja richardsoni" OR "Raja richardsoni" OR "Bathyraja scaphiops" OR "Raja scaphiops" OR "Bathyraja schroederi" OR "Breviraja schroederi" OR "Bathyraja shuntovi" OR "Bathyraja smirnovi" OR "Raja smirnovi" OR "Bathyraja smithii" OR "Raja smithii" OR "Bathyraja spinicauda" OR "Raja spinicauda" OR "Bathyraja spinosissima" OR "Psammobatis spinosissima" OR "Bathyraja taranetzi" OR "Bathyraja hubbsi" OR "Rhinoraja longi" OR "Rhinoraja taranetzi" OR "Bathyraja trachouros" OR "Breviraja trachouros" OR "Bathyraja trachura" OR "Raja microtrachys" OR "Raja trachura" OR "Bathyraja tunae" OR "Bathyraja tzinovskii" OR "Bathyraja violacea" OR "Raja violacea" OR "Bathytoshia brevicaudata" OR "Dasyatis matsubarai" OR "Dasyatis brevicaudata" OR "Dasyatis multispinosa" OR "Trygon brevicaudata" OR "Bathytoshia centroura" OR "Dasyatis centroura" OR "Dasyatis hastata" OR "Raja centroura" OR "Bathytoshia lata" OR "Dasyatis lata" OR "Dasyatis thetidis" OR "Dasyatis ushiei" OR "Trygon lata" OR "Benthobatis kreffti" OR "Benthobatis marcida" OR "Benthobatis moresbyi" OR "Benthobatis yangi" OR "Beringraja binoculata" OR "Raja cooperi" OR "Dipturus binoculata" OR "Raja binoculata" OR "Beringraja cortezensis" OR "Raja cortezensis" OR "Beringraja inornata" OR "Raja jordani" OR "Raja inornata" OR "Beringraja inornata" OR "Beringraja pulchra" OR "Raja pulchra" OR "Beringraja rhina" OR "Raia rhina" OR "Raja rhina" OR "Beringraja stellulata" OR "Raja stellulata" OR "Beringraja stellulata" OR "Breviraja claramaculata" OR "Breviraja colesi" OR "Breviraja mouldi" OR "Breviraja nigriventralis" OR "Breviraja spinosa" OR "Brevitrygon heterura" OR "Trygon heterurus" OR "Brevitrygon imbricata" OR "Himantura dadong" OR "Himantura imbricata" OR "Amphotistius imbricatus" OR "Dasyatis imbricata" OR "Dasybatus imbricatus" OR "Raja imbricata" OR "Trygon imbricata" OR "Leiobatis polylepis" OR "Raja obtusa" OR "Brevitrygon javaensis" OR "Himantura javaensis" OR "Brevitrygon manjajiae" OR "Brevitrygon walga" OR "Himantura walga" OR "Trygon walga" OR "Brochiraja aenigma" OR "Brochiraja albilabiata" OR "Brochiraja asperula" OR "Notoraja asperula" OR "Pavoraja asperula" OR "Bathyraja asperula" OR "Brochiraja heuresa" OR "Brochiraja leviveneta" OR "Brochiraja microspinifera" OR "Brochiraja spinifera" OR "Notoraja spinifera" OR "Pavoraja spinifera" OR "Bathyraja spinifera" OR "Brochiraja vittacauda" OR "Cruriraja andamanica" OR "Raia andamanica" OR "Cruriraja atlantis" OR "Cruriraja cadenati" OR "Cruriraja durbanensis" OR "Raia durbanensis" OR "Cruriraja hulleyi" OR "Cruriraja parcomaculata" OR "Cruriraja triangularis" OR "Raia parcomaculata" OR "Cruriraja poeyi" OR "Cruriraja rugosa" OR "Dactylobatus armatus" OR "Dactylobatus clarkii" OR "Raja clarkii" OR "Dasyatis chrysonota" OR "Trygon chrysonata" OR "Dasyatis hypostigma" OR "Dasyatis hipostigma" OR "Dasyatis marmorata" OR "Dasyatis chrysonota" OR "Trygon pastinaca" OR "Dasyatis pastinaca" OR "Dasyatis pastinaca" OR "Raja pastinaca" OR "Trygon pastinaca" OR "Pastinaca laevis" OR "Pastinaca olivacea" OR "Raia pastinaca" OR "Dasyatis ujo" OR "Trygon vulgaris" OR "Dasyatis tortonesei" OR "Dentiraja australis" OR "Raja australis" OR "Dipturus australis" OR "Dentiraja cerva" OR "Raja cerva" OR "Dipturus cerva" OR "Dentiraja confusa" OR "Dipturus confusus" OR "Dentiraja endeavouri" OR "Dipturus endeavouri" OR "Dentiraja falloarga" OR "Dipturus falloargus" OR "Dentiraja flindersi" OR "Dentiraja healdi" OR "Dipturus healdi" OR "Dentiraja lemprieri" OR "Raja lemprieri" OR "Dentiraja oculus" OR "Dipturus oculus" OR "Dentiraja oculus" OR "Dentiraja polyommata" OR "Raja polyommata" OR "Dipturus polyommata" OR "Diplobatis colombiensis" OR "Diplobatis guamachensis" OR "Diplobatis ommata" OR "Discopyge ommata" OR "Diplobatis picta" OR "Diplobatus pictus" OR "Diplobatis altenai" OR "Dipturus acrobelus" OR "Dipturus amphispinus" OR "Dipturus apricus" OR "Dipturus argentinensis" OR "Zearaja argentinensis" OR "Dipturus batis" OR "Raja batis" OR "Raia flossada" OR "Raia gaimardi" OR "Propterygia hyposticta" OR "Raja macrorynchus" OR "Batis vulgaris" OR "Raia vulgaris" OR "Dipturus brevicaudatus" OR "Raia brevicaudata" OR "Dipturus brevicaudata" OR "Dipturus bullisi" OR "Raja bullisi" OR "Dipturus campbelli" OR "Raja campbelli" OR "Dipturus canutus" OR "Dipturus chilensis" OR "Raia chilensis" OR "Raja flavirostris" OR "Raja latastei" OR "Raja oxyptera" OR "Zearaja chilensis" OR "Dipturus chinensis" OR "Raia chinensis" OR "Dipturus crosnieri" OR "Raja crosnieri" OR "Dipturus doutrei" OR "Raja doutrei" OR "Dipturus ecuadoriensis" OR "Raja steindachneri" OR "Raja ecuadoriensis" OR "Dipturus garricki" OR "Raja garricki" OR "Dipturus gigas" OR "Raja gigas" OR "Dipturus grahamorum" OR "Dipturus grahami" OR "Dipturus gudgeri" OR "Dipturus innominatus" OR "Raja innominata" OR "Dipturus intermedius" OR "Dipturus intermedia" OR "Raia intermedia" OR "Dipturus johannisdavisi" OR "Raja johannisdavisi" OR "Dipturus johannisdavesi" OR "Dipturus kwangtungensis" OR "Raja kwangtungensis" OR "Dipturus laevis" OR "Raja laevis" OR "Raia granulata" OR "Dipturus lamillai" OR "Dipturus lanceorostratus" OR "Raja lanceorostrata" OR "Dipturus leptocaudus" OR "Raja leptocauda" OR "Dipturus macrocauda" OR "Dipturus macrocaudus" OR "Raja macrocauda" OR "Dipturus maugeanus" OR "Dipturus melanospilus" OR "Dipturus mennii" OR "Dipturus diehli" OR "Dipturus nasutus" OR "Dipturus nasutus" OR "Raja nasuta" OR "Zearaja nasutus" OR "Dipturus nidarosiensis" OR "Raja nidarosiensis" OR "Dipturus olseni" OR "Raja olseni" OR "Dipturus oregoni" OR "Raja oregoni" OR "Dipturus oxyrinchus" OR "Raja oxyrinchus" OR "Dipturus pullopunctatus" OR "Raja pullopunctata" OR "Dipturus queenslandicus" OR "Dipturus springeri" OR "Raja springeri" OR "Dipturus stenorhynchus" OR "Raja stenorhynchus" OR "Dipturus teevani" OR "Raja teevani" OR "Dipturus tengu" OR "Raja tengu" OR "Dipturus trachydermus" OR "Raja trachyderma" OR "Dipturus trachyderma" OR "Dipturus wengi" OR "Dipturus wuhanlingi" OR "Discopyge castelloi" OR "Discopyge tschudii" OR "Electrolux addisoni" OR "Fenestraja atripinna" OR "Breviraja atripinna" OR "Fenestraja cubensis" OR "Breviraja cubensis" OR "Fenestraja ishiyamai" OR "Breviraja ishiyamai" OR "Fenestraja maceachrani" OR "Gurgesiella maceachrani" OR "Fenestraja mamillidens" OR "Raja mamillidens" OR "Fenestraja plutonia" OR "Raja plutonia" OR "Fenestraja sibogae" OR "Gurgesiella sibogae" OR "Raja sibogae" OR "Fenestraja sinusmexicanus" OR "Breviraja sinusmexicanus" OR "Fluvitrygon kittipongi" OR "Himantura kittipongi" OR "Fluvitrygon oxyrhynchus" OR "Himantura oxyrhynchus" OR "Himantura oxyrhyncha" OR "Trygon oxyrhynchus" OR "Himantura krempfi" OR "Fluvitrygon signifer" OR "Himantura signifer" OR "Fontitrygon colarensis" OR "Dasyatis colarensis" OR "Fontitrygon garouaensis" OR "Dasyatis garouaensis" OR "Potamotrygon garouaensis" OR "Fontitrygon geijskesi" OR "Dasyatis geijskesi" OR "Fontitrygon margarita" OR "Dasyatis margarita" OR "Trygon margarita" OR "Fontitrygon margaritella" OR "Dasyatis margaritella" OR "Fontitrygon ukpam" OR "Dasyatis ukpam" OR "Urogymnus ukpam" OR "Hemitrygon ukpam" OR "Glaucostegus cemiculus" OR "Rhinobatos cemiculus" OR "Glaucostegus petiti" OR "Glaucostegus granulatus" OR "Rhinobatos granulatus" OR "Glaucostegus halavi" OR "Rhinobatos halavi" OR "Raja halavi" OR "Glaucostegus obtusus" OR "Rhinobatos obtusus" OR "Glaucostegus thouin" OR "Rhinobatos thouin" OR "Raja thouin" OR "Glaucostegus typus" OR "Glaucostegus microphthalmus" OR "Rhinobatos typus" OR "Glaucostegus younholeei" OR "Gurgesiella atlantica" OR "Pseudoraja atlantica" OR "Gurgesiella dorsalifera" OR "Gurgesiella furvescens" OR "Gymnura altavela" OR "Raja altavela" OR "Pteroplatea binotata" OR "Pteroplatea canariensis" OR "Raja maclura" OR "Pteroplatea vaillantii" OR "Pteroplatea valenciennii" OR "Gymnura australis" OR "Pteroplatea australis" OR "Gymnura crebripunctata" OR "Gymnura afuerae" OR "Pteroplatea crebripunctata" OR "Gymnura japonica" OR "Gymnura bimaculata" OR "Pteroplatea japonica" OR "Gymnura lessae" OR "Gymnura marmorata" OR "Pteroplatea marmorata" OR "Pteroplatea rava" OR "Gymnura micrura" OR "Raja micrura" OR "Gymnura natalensis" OR "Pteroplatea natalensis" OR "Gymnura poecilura" OR "Gymnura sereti" OR "Gymnura tentaculata" OR "Aetoplatea tentaculata" OR "Gymnura zonura" OR "Aetoplatea zonura" OR "Heliotrygon rosai" OR "Hemitrygon akajei" OR "Dasyatis akajei" OR "Dasyatis gigantea" OR "Trygon akajei" OR "Hemitrygon bennetti" OR "Dasyatis bennetti" OR "Trygon bennettii" OR "Hemitrygon fluviorum" OR "Dasyatis fluviorum" OR "Hemitrygon izuensis" OR "Dasyatis izuensis" OR "Hemitrygon laevigata" OR "Dasyatis laevigata" OR "Hemitrygon laosensis" OR "Dasyatis laosensis" OR "Hemitrygon longicauda" OR "Dasyatis longicauda" OR "Hemitrygon navarrae" OR "Dasyatis navarrae" OR "Trygon navarrae" OR "Hemitrygon parvonigra" OR "Dasyatis parvonigra" OR "Hemitrygon sinensis" OR "Dasyatis sinensis" OR "Trygon sinensis" OR "Hemitrygon yemenensis" OR "Heteronarce bentuviai" OR "Narcine bentuviai" OR "Heteronarce garmani" OR "Heteronarce regani" OR "Narcine natalensis" OR "Heteronarce mollis" OR "Narcine mollis" OR "Hexatrygon bickelli" OR "Hexatrygon yangi" OR "Hexatrematobatis longirostrum" OR "Hexatrygon taiwanensis" OR "Hexatrygon brevirostra" OR "Himantura australis" OR "Himantura leoparda" OR "Trygon russellii" OR "Himantura uarnak" OR "Raja uarnak" OR "Himantura punctata" OR "Himantura variegatus" OR "Raja sephen" OR "Himantura undulata" OR "Hongeo koreana" OR "Okamejei koreana" OR "Raja koreana" OR "Hypanus americanus" OR "Dasyatis americana" OR "Hypanus berthalutzae" OR "Hypanus dipterurus" OR "Dasyatis brevis" OR "Dasyatis hawaiiensis" OR "Dasyatis dipterura" OR "Dasybatis dipterurus" OR "Hypanus guttatus" OR "Dasyatis guttata" OR "Raja guttata" OR "Hypanus longus" OR "Dasyatis longus" OR "Dasyatis longa" OR "Trygon longa" OR "Hypanus marianae" OR "Dasyatis marianae" OR "Hypanus rudis" OR "Dasyatis rudis" OR "Trygon rudis" OR "Hypanus sabinus" OR "Dasyatis sabina" OR "Trygon sabina" OR "Hypanus say" OR "Dasyatis sayi" OR "Dasyatis say" OR "Raja say" OR "Hypnos monopterygius" OR "Indobatis ori" OR "Springeria ori" OR "Anacanthobatis ori" OR "Insentiraja laxipella" OR "Notoraja laxipella" OR "Pavoraja laxipella" OR "Insentiraja subtilispinosa" OR "Notoraja subtilispinosa" OR "Irolita waitii" OR "Psammobatis waitii" OR "Raja waitii" OR "Irolita westraliensis" OR "Leucoraja circularis" OR "Raja circularis" OR "Raja falsavela" OR "Leucoraja compagnoi" OR "Raja compagnoi" OR "Leucoraja elaineae" OR "Leucoraja erinacea" OR "Raja erinaceus" OR "Leucoraja fullonica" OR "Raja fullonica" OR "Raja chagrinea" OR "Raja gallica" OR "Leucoraja garmani" OR "Raja garmani" OR "Leucoraja lentiginosa" OR "Raja lentiginosa" OR "Leucoraja leucosticta" OR "Raja leucosticta" OR "Leucoraja melitensis" OR "Leucoraja naevus" OR "Raja naevus" OR "Leucoraja ocellata" OR "Raja ocellata" OR "Leucoraja pristispina" OR "Leucoraja wallacei" OR "Raja wallacei" OR "Leucoraja yucatanensis" OR "Breviraja yucatanensis" OR "Maculabatis ambigua" OR "Maculabatis arabica" OR "Maculabatis astra" OR "Himantura astra" OR "Maculabatis bineeshi" OR "Maculabatis gerrardi" OR "Himantura macrurus" OR "Himantura alcockii" OR "Himantura gerrardi" OR "Trygon gerrardi" OR "Maculabatis macrura" OR "Trygon macrurus" OR "Maculabatis pastinacoides" OR "Himantura pareh" OR "Himantura pastinacoides" OR "Trygon pastinacoides" OR "Maculabatis randalli" OR "Himantura randalli" OR "Maculabatis toshi" OR "Himantura toshi" OR "Makararaja chindwinensis" OR "Malacoraja kreffti" OR "Raja kreffti" OR "Malacoraja obscura" OR "Malacoraja senta" OR "Raja senta" OR "Malacoraja spinacidermis" OR "Megatrygon microps" OR "Dasyatis microps" OR "Trygon microps" OR "Mobula alfredi" OR "Manta alfredi" OR "Ceratoptera alfredi" OR "Mobula birostris" OR "Manta hamiltoni" OR "Manta birostris" OR "Raja birostris" OR "Mobula eregoodoo" OR "Mobula eregoodootenkee" OR "Dicerobatis eregoodoo" OR "Mobula hypostoma" OR "Mobula rochebrunei" OR "Mobula kuhlii" OR "Cephaloptera kuhlii" OR "Dicerobatis draco" OR "Mobula mobular" OR "Raia mobular" OR "Mobula japanica" OR "Mobula munkiana" OR "Mobula tarapacana" OR "Cephaloptera tarapacana" OR "Mobula thurstoni" OR "Dicerobatis thurstoni" OR "Myliobatis aquila" OR "Raja aquila" OR "Myliobatis californicus" OR "Myliobatis californica" OR "Rhinoptera encenadae" OR "Rhinoptera vespertilio" OR "Myliobatis chilensis" OR "Myliobatis freminvillei" OR "Myliobatis freminvillii" OR "Myliobatis goodei" OR "Myliobatis hamlyni" OR "Myliobatis longirostris" OR "Myliobatis peruvianus" OR "Myliobatis ridens" OR "Myliobatis tenuicaudatus" OR "Myliobatis australis" OR "Myliobatis tobijei" OR "Myliobatis cornuta" OR "Narcine atzi" OR "Narcine baliensis" OR "Narcine bancroftii" OR "Torpedo bancroftii" OR "Narcine brasiliensis" OR "Torpedo brasiliensis" OR "Narcine brevilabiata" OR "Narcine entemedor" OR "Narcine insolita" OR "Narcine leoparda" OR "Narcine lingula" OR "Narcine maculata" OR "Raja maculata" OR "Narcine oculifera" OR "Narcine prodorsalis" OR "Narcine rierai" OR "Heteronarce rierai" OR "Narcine timlei" OR "Narcine indica" OR "Raja timlei" OR "Narcine vermiculata" OR "Narcine schmidti" OR "Narcine vermiculatus" OR "Narcinops lasti" OR "Narcine lasti" OR "Narcinops nelsoni" OR "Narcine nelsoni" OR "Narcinops ornata" OR "Narcine ornata" OR "Narcinops tasmaniensis" OR "Narcine tasmaniensis" OR "Narcinops westraliensis" OR "Narcine westraliensis" OR "Narke capensis" OR "Raja capensis" OR "Narke dipterygia" OR "Raja dipterygia" OR "Bengalichthys impennis" OR "Narke japonica" OR "Torpedo japonica" OR "Crassinarke dormitor" OR "Neoraja africana" OR "Breviraja africana" OR "Neoraja caerulea" OR "Breviraja caerulea" OR "Neoraja carolinensis" OR "Neoraja iberica" OR "Neoraja stehmanni" OR "Breviraja stehmanni" OR "Neotrygon annotata" OR "Dasyatis annotata" OR "Dasyatis annotatus" OR "Neotrygon australiae" OR "Neotrygon caeruleopunctata" OR "Neotrygon kuhlii" OR "Amphotistius kuhlii" OR "Dasyatis kuhlii" OR "Trygon kuhlii" OR "Neotrygon leylandi" OR "Dasyatis leylandi" OR "Neotrygon ningalooensis" OR "Neotrygon orientalis" OR "Neotrygon orientale" OR "Neotrygon picta" OR "Neotrygon trigonoides" OR "Raya trigonoides" OR "Neotrygon varidens" OR "Dasybatus varidens" OR "Notoraja alisae" OR "Notoraja azurea" OR "Notoraja fijiensis" OR "Notoraja hesperindica" OR "Notoraja hirticauda" OR "Notoraja inusitata" OR "Notoraja lira" OR "Notoraja longiventralis" OR "Notoraja martinezi" OR "Notoraja ochroderma" OR "Notoraja sapphira" OR "Notoraja sereti" OR "Notoraja sticta" OR "Notoraja tobitukai" OR "Raja tobitukai" OR "Okamejei acutispina" OR "Raja acutispina" OR "Okamejei arafurensis" OR "Okamejei boesemani" OR "Raja boesemani" OR "Okamejei cairae" OR "Okamejei heemstrai" OR "Raja heemstrai" OR "Okamejei hollandi" OR "Raja hollandi" OR "Okamejei kenojei" OR "Raja kenojei" OR "Okamejei leptoura" OR "Okamejei meerdervoortii" OR "Raja meerdervoortii" OR "Okamejei mengae" OR "Okamejei ornata" OR "Okamejei panayensis" OR "Okamejei picta" OR "Okamejei schmidti" OR "Raja schmidti" OR "Orbiraja jensenae" OR "Okamejei jensenae" OR "Orbiraja philipi" OR "Raia philipi" OR "Okamejei powelli" OR "Okamejei philipi" OR "Orbiraja powelli" OR "Okamejei powelli" OR "Raja powelli" OR "Paratrygon aiereba" OR "Paratrygon ajereba" OR "Trygon ajereba" OR "Raja orbicularis" OR "Pastinachus ater" OR "Pastinachus atrus" OR "Pastinachus sephen" OR "Taeniura atra" OR "Pastinachus gracilicaudus" OR "Pastinachus sephen" OR "Dasyatis sephen" OR "Hypolophus sephen" OR "Raja sephen" OR "Himantura fluviatilis" OR "Pastinachus solocirostris" OR "Pastinachus stellurostris" OR "Pateobatis bleekeri" OR "Himantura bleekeri" OR "Trygon bleekeri" OR "Pateobatis fai" OR "Himantura fai" OR "Pateobatis hortlei" OR "Himantura hortlei" OR "Pateobatis jenkinsii" OR "Himantura draco" OR "Himantura jenkinsii" OR "Trygon jenkinsii" OR "Pateobatis uarnacoides" OR "Himantura uarnacoides" OR "Trygon uarnacoides" OR "Pavoraja alleni" OR "Pavoraja arenaria" OR "Pavoraja mosaica" OR "Pavoraja nitida" OR "Pavoraja pseudonitida" OR "Pavoraja umbrosa" OR "Platyrhina hyugaensis" OR "Platyrhina psomadakisi" OR "Platyrhina sinensis" OR "Rhina sinensis" OR "Platyrhina limboonkengi" OR "Platyrhina tangi" OR "Platyrhinoidis triseriata" OR "Platyrhina triseriata" OR "Plesiobatis daviesi" OR "Urolophus marmoratus" OR "Plesiotrygon iwamae" OR "Plesiotrygon nana" OR "Pliotrema annae" OR "Pliotrema kajae" OR "Pliotrema warreni" OR "Potamotrygon adamastor" OR "Potamotrygon amandae" OR "Potamotrygon amazona" OR "Potamotrygon boesemani" OR "Potamotrygon brachyura" OR "Trygon brachyurus" OR "Potamotrygon brumi" OR "Potamotrygon castexi" OR "Potamotrygon constellata" OR "Taeniura constellata" OR "Potamotrygon falkneri" OR "Potamotrygon menchacai" OR "Potamotrygon castexi" OR "Potamotrygon henlei" OR "Trygon henlei" OR "Taenura henlei" OR "Potamotrygon histrix" OR "Trygon hystrix" OR "Potamotrygon humerosa" OR "Potamotrygon orbignyi" OR "Potamotrygon leopoldi" OR "Potamotrygon limai" OR "Potamotrygon magdalenae" OR "Trygon hystrix" OR "Taeniura magdalenae" OR "Potamotrygon marinae" OR "Potamotrygon motoro" OR "Potamotrygon pauckei" OR "Taeniura motoro" OR "Potamotrygon alba" OR "Trygon garrapa" OR "Potamotrygon labradori" OR "Potamotrygon laticeps" OR "Potamotrygon ocellata" OR "Trygon ocellata" OR "Potamotrygon orbignyi" OR "Potamotrygon dumerilii" OR "Trygon orbignyi" OR "Potamotrygon reticulatus" OR "Potamotrygon roulini" OR "Potamotrygon rex" OR "Potamotrygon schroederi" OR "Potamotrygon schuemacheri" OR "Potamotrygon scobina" OR "Potamotrygon signata" OR "Potamotrygon tigrina" OR "Potamotrygon yepezi" OR "Pristiophorus cirratus" OR "Pristis cirratus" OR "Pristiophorus delicatus" OR "Pristiophorus japonicus" OR "Pristiophorus lanae" OR "Pristiophorus nancyae" OR "Pristiophorus nudipinnis" OR "Pristiophorus schroederi" OR "Pristis clavata" OR "Pristis pectinata" OR "Pristis acutirostris" OR "Pristis annandalei" OR "Pristis granulosa" OR "Pristis mississippiensis" OR "Pristis serra" OR "Pristis pristis" OR "Pristis zephyreus" OR "Pristis perotteti" OR "Pristis microdon" OR "Squalus pristis" OR "Pristis zijsron" OR "Psammobatis bergi" OR "Psammobatis extenta" OR "Psammobatis glansdissimilis" OR "Raia extenta" OR "Psammobatis lentiginosa" OR "Psammobatis normani" OR "Psammobatis parvacauda" OR "Psammobatis rudis" OR "Psammobatis rutrum" OR "Malacorhina cirrifer" OR "Psammobatis scobina" OR "Raja scobina" OR "Pseudobatos buthi" OR "Pseudobatos glaucostigmus" OR "Rhinobatos glaucostigma" OR "Pseudobatos horkelii" OR "Rhinobatos horkeli" OR "Rhinobatos horkelii" OR "Pseudobatos lentiginosus" OR "Rhinobatos lentiginosus" OR "Pseudobatos leucorhynchus" OR "Rhinobatos leucorhynchus" OR "Rhinobatos leucorhinchus" OR "Pseudobatos percellens" OR "Rhinobatos percellens" OR "Raja percellens" OR "Pseudobatos planiceps" OR "Rhinobatos planiceps" OR "Pseudobatos prahli" OR "Rhinobatos prahli" OR "Pseudobatos productus" OR "Rhinobatos productus" OR "Pseudoraja fischeri" OR "Pteroplatytrygon violacea" OR "Dasyatis violacea" OR "Trygon violacea" OR "Raja asterias" OR "Raja brachyura" OR "Raia brachyura" OR "Raja clavata" OR "Malacoraja clavata" OR "Raja herwigi" OR "Raja maderensis" OR "Raia maderensis" OR "Raja microocellata" OR "Raia microocellata" OR "Raja miraletus" OR "Raja montagui" OR "Raja ocellifera" OR "Raja parva" OR "Raja pita" OR "Okamejei pita" OR "Raja polystigma" OR "Raja radula" OR "Raja straeleni" OR "Raja rouxi" OR "Raja undulata" OR "Raja fenestrata" OR "Raja atra" OR "Raja mosaica" OR "Raja picta" OR "Rajella annandalei" OR "Raja annandalei" OR "Rajella barnardi" OR "Raja confundens" OR "Raja dageti" OR "Raja barnardi" OR "Rajella bathyphila" OR "Raja bathyphila" OR "Rajella bigelowi" OR "Raja bigelowi" OR "Rajella caudaspinosa" OR "Raia caudaspinosa" OR "Rajella challengeri" OR "Rajella dissimilis" OR "Rajella eisenhardti" OR "Rajella fuliginea" OR "Raja fuliginea" OR "Rajella fyllae" OR "Breviraja marklei" OR "Breviraja mouldi" OR "Raja fyllae" OR "Rajella kukujevi" OR "Raja kukujevi" OR "Rajella leoparda" OR "Rajella leopardus" OR "Raia leopardus" OR "Rajella lintea" OR "Dipturus linteus" OR "Raja lintea" OR "Rajella nigerrima" OR "Malacoraja nigerrima" OR "Breviraja nigerrima" OR "Rajella paucispinosa" OR "Rajella purpuriventralis" OR "Raja purpuriventralis" OR "Rajella ravidula" OR "Raja ravidula" OR "Rajella sadowskii" OR "Raja sadowskii" OR "Rhina ancylostomus" OR "Rhina ancylostoma" OR "Rhinobatos albomaculatus" OR "Rhinobatos annandalei" OR "Rhinobatos austini" OR "Rhinobatos borneensis" OR "Rhinobatos holcorhynchus" OR "Rhinobatos hynnicephalus" OR "Rhinobatos irvinei" OR "Rhinobatos jimbaranensis" OR "Rhinobatos lionotus" OR "Rhinobatos manai" OR "Rhinobatos nudidorsalis" OR "Rhinobatos penggali" OR "Rhinobatos punctifer" OR "Rhinobatos ranongensis" OR "Rhinobatos rhinobatos" OR "Raja rhinobatos" OR "Rhinobatos sainsburyi" OR "Rhinobatos schlegelii" OR "Rhinobatos formosensis" OR "Rhinobatos whitei" OR "Rhinoptera bonasus" OR "Raja bonasus" OR "Rhinoptera brasiliensis" OR "Rhinoptera javanica" OR "Rhinoptera jayakari" OR "Rhinoptera marginata" OR "Myliobatis marginata" OR "Rhinoptera neglecta" OR "Rhinoptera steindachneri" OR "Rhinoraja kujiensis" OR "Bathyraja kujiensis" OR "Rhinoraja longicauda" OR "Bathyraja longicauda" OR "Rhinoraja odai" OR "Bathyraja odai" OR "Rhynchobatus australiae" OR "Rhynchobatus djiddensis" OR "Rhynchobatus cooki" OR "Rhynchobatus djiddensis" OR "Raja djiddensis" OR "Rhynchobatus immaculatus" OR "Rhynchobatus laevis" OR "Rhinobatus laevis" OR "Rhynchobatus luebberti" OR "Rhynchobatus atlanticus" OR "Rhynchobatus mononoke" OR "Rhynchobatus palpebratus" OR "Rhynchobatus springeri" OR "Rhynchorhina mauritaniensis" OR "Rioraja agassizii" OR "Uraptera agassizii" OR "Rostroraja ackleyi" OR "Raja ackleyi" OR "Rostroraja alba" OR "Raja marginata" OR "Raja alba" OR "Rostroraja bahamensis" OR "Raja bahamensis" OR "Rostroraja cervigoni" OR "Raja cervigoni" OR "Rostroraja eglanteria" OR "Raja eglanteria" OR "Rostroraja equatorialis" OR "Raja equatorialis" OR "Rostroraja texana" OR "Raja texana" OR "Rostroraja velezi" OR "Raja velezi" OR "Schroederobatis americana" OR "Anacanthobatis americanus" OR "Sinobatis andamanensis" OR "Sinobatis borneensis" OR "Anacanthobatis borneensis" OR "Anacanthobatis donghaiensis" OR "Anacanthobatis nanhaiensis" OR "Sinobatis brevicauda" OR "Sinobatis bulbicauda" OR "Sinobatis caerulea" OR "Sinobatis filicauda" OR "Sinobatis kotlyari" OR "Sinobatis melanosoma" OR "Anacanthobatis melanosoma" OR "Springeria melanosoma" OR "Sinobatis stenosoma" OR "Anacanthobatis stenosoma" OR "Springeria stenosoma" OR "Spinilophus armatus" OR "Urolophus armatus" OR "Spiniraja whitleyi" OR "Dipturus whitleyi" OR "Raja whitleyi" OR "Springeria folirostris" OR "Anacanthobatis folirostris" OR "Springeria longirostris" OR "Anacanthobatis longirostris" OR "Styracura pacifica" OR "Dasyatis pacificus" OR "Himantura pacifica" OR "Styracura schmardae" OR "Trygon schmardae" OR "Himantura schmardae" OR "Sympterygia acuta" OR "Raja echinorhyncha" OR "Sympterygia bonapartii" OR "Sympterygia bonapartei" OR "Raia marplatensis" OR "Sympterygia brevicaudata" OR "Psammobatis brevicaudatus" OR "Psammobatis asper" OR "Psammobatis caudispina" OR "Psammobatis chilcae" OR "Sympterygia lima" OR "Raja lima" OR "Raja acanthostyla" OR "Raja buergeri" OR "Raja chilensis" OR "Raja steindachneri" OR "Raja steindachneri" OR "Taeniura lessoni" OR "Taeniura lymma" OR "Raja lymma" OR "Taeniurops grabatus" OR "Trygon grabatus" OR "Taeniura grabata" OR "Taeniurops grabata" OR "Taeniurops meyeni" OR "Taeniura melanospilos" OR "Taeniura meyeni" OR "Telatrygon acutirostra" OR "Dasyatis acutirostra" OR "Telatrygon biasa" OR "Telatrygon crozieri" OR "Trygon crozieri" OR "Telatrygon zugei" OR "Dasyatis zugei" OR "Trygon zugei" OR "Temera hardwickii" OR "Tetronarce californica" OR "Torpedo californica" OR "Tetronarce cowleyi" OR "Tetronarce formosa" OR "Torpedo formosa" OR "Tetronarce nobiliana" OR "Tetronarce fairchildi" OR "Tetronarce macneilli" OR "Torpedo nobiliana" OR "Tetronarce occidentalis" OR "Torpedo occidentalis" OR "Tetronarce puelcha" OR "Torpedo puelcha" OR "Tetronarce tokionis" OR "Torpedo tokionis" OR "Tetronarcine tokionis" OR "Tetronarce tremens" OR "Torpedo tremens" OR "Torpedo peruana" OR "Torpedo microdiscus" OR "Torpedo adenensis" OR "Torpedo andersoni" OR "Torpedo bauchotae" OR "Torpedo fuscomaculata" OR "Torpedo mackayana" OR "Torpedo marmorata" OR "Torpedo panthera" OR "Torpedo sinuspersici" OR "Torpedo suessii" OR "Torpedo torpedo" OR "Torpedo ocellata" OR "Raja torpedo" OR "Trygonoptera galba" OR "Trygonoptera imitata" OR "Trygonoptera mucosa" OR "Trygonoptera ovalis" OR "Trygonoptera personata" OR "Trygonoptera testacea" OR "Trygonorrhina dumerilii" OR "Trygonorrhina melaleuca" OR "Trygonorrhina fasciata" OR "Trygonorrhina spA" OR "Typhlonarke aysoni" OR "Typhlonarke tarakea" OR "Astrape aysoni" OR "Urobatis concentricus" OR "Urobatis halleri" OR "Urolophus halleri" OR "Urobatis jamaicensis" OR "Urolophus jamaicensis" OR "Urobatis maculatus" OR "Urobatis marmoratus" OR "Urolophus marmoratus" OR "Urobatis pardalis" OR "Urobatis tumbesensis" OR "Urolophus tumbesensis" OR "Urogymnus acanthobothrium" OR "Urogymnus asperrimus" OR "Raja asperrima" OR "Urogymnus dalyensis" OR "Himantura dalyensis" OR "Urogymnus granulatus" OR "Himantura ponapensis" OR "Himantura granulata" OR "Trygon granulata" OR "Urogymnus lobistoma" OR "Himantura lobistoma" OR "Urogymnus lobistomus" OR "Urogymnus polylepis" OR "Himantura chaophraya" OR "Himantura polylepis" OR "Trygon polylepis" OR "Urogymnus laevior" OR "Urolophus aurantiacus" OR "Urolophus bucculentus" OR "Urolophus circularis" OR "Urolophus cruciatus" OR "Raja cruciata" OR "Urolophus deforgesi" OR "Urolophus expansus" OR "Urolophus flavomosaicus" OR "Urolophus gigas" OR "Urolophus javanicus" OR "Trygonoptera javanica" OR "Urolophus kaianus" OR "Urolophus kapalensis" OR "Urolophus lobatus" OR "Urolophus mitosis" OR "Urolophus neocaledoniensis" OR "Urolophus orarius" OR "Urolophus papilio" OR "Urolophus paucimaculatus" OR "Urolophus piperatus" OR "Urolophus sufflavus" OR "Urolophus viridis" OR "Urolophus westraliensis" OR "Urotrygon aspidura" OR "Urolophus aspidurus" OR "Urotrygon chilensis" OR "Urolophus chilensis" OR "Urolophus goodei" OR "Urotrygon cimar" OR "Urotrygon microphthalmum" OR "Urotrygon munda" OR "Urotrygon nana" OR "Urotrygon reticulata" OR "Urotrygon rogersi" OR "Urolophus rogersi" OR "Urotrygon simulatrix" OR "Urotrygon venezuelae" OR "Zanobatus maculatus" OR "Zanobatus schoenleinii" OR "Platyrhina schoenleinii" OR "Zapteryx brevirostris" OR "Rhinobatus brevirostris" OR "Zapteryx exasperata" OR "Platyrhina exasperata" OR "Zapteryx xyster")*

***References***

Bland, L.M., Bielby, J., Kearney, S., Orme, C.D.L., Watson, J.E.M., Collen, B., 2017. Toward reassessing data‐deficient species. Conserv. Biol. 31, 531–539. https://doi.org/10.1111/cobi.12850

Clark, J.A., May, R.M., 2002. Taxonomic Bias in Conservation Research. Science 297, 191–192. https://doi.org/10.1126/science.297.5579.191b

Costello, M.J., Lane, M., Wilson, S., Houlding, B., 2015. Factors influencing when species are first named and estimating global species richness. Glob. Ecol. Conserv. 4, 243–254. https://doi.org/10.1016/j.gecco.2015.07.001

de Oliveira, C.D.L., Ladle, R.J., da Silva Batista, V., 2023. Patterns and trends in scientific production on marine elasmobranchs: research hotspots and emerging themes for conservation. J. Coast. Conserv. 27, 6. https://doi.org/10.1007/s11852-023-00937-z

Dos Santos, J.W., Correia, R.A., Malhado, A.C.M., Campos‐Silva, J.V., Teles, D., Jepson, P., Ladle, R.J., 2020. Drivers of taxonomic bias in conservation research: a global analysis of terrestrial mammals. Anim. Conserv. 23, 679–688. https://doi.org/10.1111/acv.12586

Dulvy, N.K., Pacoureau, N., Matsushiba, J.H., Yan, H.F., VanderWright, W.J., Rigby, C.L., Finucci, B., Sherman, C.S., Jabado, R.W., Carlson, J.K., Pollom, R.A., Charvet, P., Pollock, C.M., Hilton-Taylor, C., Simpfendorfer, C.A., 2024. Ecological erosion and expanding extinction risk of sharks and rays. Science 386. https://doi.org/10.1126/science.adn1477

Dulvy, N.K., Pacoureau, N., Rigby, C.L., Pollom, R.A., Jabado, R.W., Ebert, D.A., Finucci, B., Pollock, C.M., Cheok, J., Derrick, D.H., Herman, K.B., Sherman, C.S., VanderWright, W.J., Lawson, J.M., Walls, R.H.L., Carlson, J.K., Charvet, P., Bineesh, K.K., Fernando, D., Ralph, G.M., Matsushiba, J.H., Hilton-Taylor, C., Fordham, S.V., Simpfendorfer, C.A., 2021. Overfishing drives over one-third of all sharks and rays toward a global extinction crisis. Curr. Biol. 31, 4773-4787.e8. https://doi.org/10.1016/j.cub.2021.08.062

Guedes, J.J.M., Moura, M.R., Alexandre F. Diniz‐Filho, J., 2023. Species out of sight: elucidating the determinants of research effort in global reptiles. Ecography 2023, e06491. https://doi.org/10.1111/ecog.06491

Guénard, B., Hughes, A.C., Lainé, C., Cannicci, S., Russell, B.D., Williams, G.A., 2025. Limited and biased global conservation funding means most threatened species remain unsupported. Proc. Natl. Acad. Sci. 122. https://doi.org/10.1073/pnas.2412479122

Langlois, J., Guilhaumon, F., Baletaud, F., Casajus, N., De Almeida Braga, C., Fleuré, V., Kulbicki, M., Loiseau, N., Mouillot, D., Renoult, J.P., Stahl, A., Stuart Smith, R.D., Tribot, A.-S., Mouquet, N., 2022. The aesthetic value of reef fishes is globally mismatched to their conservation priorities. PLOS Biol. 20, e3001640. https://doi.org/10.1371/journal.pbio.3001640

Luiz, O.J., Allen, A.P., Robertson, D.R., Floeter, S.R., Kulbicki, M., Vigliola, L., Becheler, R., Madin, J.S., 2013. Adult and larval traits as determinants of geographic range size among tropical reef fishes. Proc. Natl. Acad. Sci. 110, 16498–16502. https://doi.org/10.1073/pnas.1304074110

Lythgoe, J.N., 1979. The Ecology of Vision, Oxford science publications. Clarendon Press.

Mammola, S., Adamo, M., Antić, D., Calevo, J., Cancellario, T., Cardoso, P., Chamberlain, D., Chialva, M., Durucan, F., Fontaneto, D., Goncalves, D., Martínez, A., Santini, L., Rubio-Lopez, I., Sousa, R., Villegas-Rios, D., Verdes, A., Correia, R.A., 2023. Drivers of species knowledge across the tree of life. eLife 12. https://doi.org/10.7554/elife.88251.3

Melnychuk, M.C., Peterson, E., Elliott, M., Hilborn, R., 2017. Fisheries management impacts on target species status. Proc. Natl. Acad. Sci. 114, 178–183. https://doi.org/10.1073/pnas.1609915114

Moura, M.R., Jetz, W., 2021. Shortfalls and opportunities in terrestrial vertebrate species discovery. Nat. Ecol. Evol. 5, 631–639. https://doi.org/10.1038/s41559-021-01411-5

Reynolds, J.D., Dulvy, N.K., Goodwin, N.B., Hutchings, J.A., 2005. Biology of extinction risk in marine fishes. Proc. R. Soc. B Biol. Sci. 272, 2337–2344. https://doi.org/10.1098/rspb.2005.3281

Smith, K.F., Brown, J.H., 2002. Patterns of diversity, depth range and body size among pelagic fishes along a gradient of depth. Glob. Ecol. Biogeogr. 11, 313–322. https://doi.org/10.1046/j.1466-822X.2002.00286.x

Strona, G., Galli, P., Montano, S., Seveso, D., Fattorini, S., 2012. Global-Scale Relationships between Colonization Ability and Range Size in Marine and Freshwater Fish. PLoS ONE 7, e49465. https://doi.org/10.1371/journal.pone.0049465

Titley, M.A., Snaddon, J.L., Turner, E.C., 2017. Scientific research on animal biodiversity is systematically biased towards vertebrates and temperate regions. PLOS ONE 12, e0189577. https://doi.org/10.1371/journal.pone.0189577
